# Optimal control of a heat flow system via novel enhanced quadratic interpolation optimization tuned 2DOF-PID controller

**DOI:** 10.1038/s41598-026-62513-3

**Published:** 2026-07-21

**Authors:** Gökhan Yüksek, Kaan Can, Serdar Ekinci, Erdal Akin

**Affiliations:** 1 Faculty of Engineering and Architecture, Department of Electrical and Electronics Engineering, Batman University, Merkez, Batman, Turkey; 2 Faculty of Engineering, Department of Electrical and Electronics Engineering, Atatürk University, Erzurum, 25240 Turkey; 3 Department of Computer Engineering, Bitlis Eren University, Bitlis, 13100 Turkey; 4 Department of Computer Science and Media Technology, Malmö University, Malmö, 205 06 Sweden

**Keywords:** Heat flow system, Two-degree-of-freedom PID controller, Enhanced Quadratic Interpolation Optimization, Metaheuristic optimization, Optimal control, Temperature regulation, Engineering, Mathematics and computing

## Abstract

This study addresses the optimal temperature regulation problem of a laboratory-scale heat flow system using a two-degree-of-freedom proportional–integral–derivative (2DOF-PID) controller tuned by a newly developed enhanced quadratic interpolation optimization (eQIO) algorithm. The main contribution lies in improving the original quadratic interpolation optimization (QIO) framework by integrating two lightweight enhancement mechanisms: a periodic parabolic local search to strengthen local exploitation and a stagnation-aware diversity recovery strategy to prevent premature convergence. The enhanced formulation preserves the deterministic interpolation-based structure of QIO while increasing convergence reliability and robustness without introducing additional algorithmic complexity. The effectiveness of eQIO is first evaluated on ten benchmark functions from the CEC-2019 test suite and compared with QIO, mountain gazelle optimization, tuned moss growth optimization, whale optimization algorithm, and superb fairy-wren optimization algorithm. Statistical results obtained from 500 independent runs indicate that eQIO consistently improves average solution quality and reduces standard deviation relative to QIO, while achieving competitive performance against population-based metaheuristic algorithms. Subsequently, eQIO is employed to tune the parameters of the 2DOF-PID controller for real-time temperature regulation experiments under step, square, and sinusoidal reference signals. The experimental results demonstrate reduced overshoot, lower steady-state error, improved tracking accuracy, and smoother control effort compared with alternative optimizer-based tuning approaches. Quantitatively, the proposed eQIO-based 2DOF-PID controller reduces the total mean absolute error (MAE) by 14.8% under step–square reference signals and by 45.8% under step–sinusoidal reference signals compared with the original QIO-based controller, while also providing smoother control action and improved tracking robustness. Overall, the proposed eQIO-driven 2DOF-PID framework establishes a computationally efficient and experimentally validated methodology for reliable thermal system control.

## Introduction

 Temperature regulation is a crucial operation, and it is a classic challenge in many engineering and industrial applications, e.g., thermal tank systems or heating, ventilation and air conditioning systems^1–3^. Such systems typically have non-linear dynamics, time delays, parametric uncertainties, and external disturbances, which render temperature control a formidable task. For applications in which continuous and precise temperature tracking is mandatory, the proper tuning of the control parameters plays an important role in achieving stability, a fast-transient response, and robustness. The incorrect selection of parameters can also result in oscillations, excessive overshoot, steady-state errors, or higher energy consumption^4^. Therefore, advanced control strategies and optimization-based tuning methods are frequently employed to enhance tracking performance, disturbance rejection capability and overall system efficiency. Extensive research has been conducted in the literature on temperature control systems to overcome these challenges and enhance temperature control performance^5,6^. To compare the effectiveness and performance of the proposed optimization methods, all methods were compared with the classical Ziegler–Nichols (Z-N) tuning method. The experimental results indicated that the temperature tracking performance achieved with the parameters determined by the DE algorithm, outperformed to the other methods. Jovanovic et al. proposed artificial neural networks and adaptive neural-fuzzy inference (ANFIS)-based non-linear methods for modelling and controlling heat transfer within a chamber^7^. To obtain a novel model valid across the entire state space, the researchers developed two different identification approaches based on feedforward artificial neural network (FFNN) and ANFIS. The results showed that both FFNN and ANFIS-based non-linear models provided high accuracy across the entire operating range and efficiently generated the control signal. As another study, Ahn et al. proposed a novel fractional-order integral derivative (FO-ID) controller in temperature profile tracking control^8^. The obtained results were experimentally compared with a conventional PI/PID designed based on the Z-N tuning method and FO-PI controllers. The obtained results demonstrated the superiority and enhanced robustness of the proposed FO-ID controller in terms of temperature tracking performance.

In recent years, research interest has increasingly shifted toward metaheuristic and swarm-based optimization techniques for tuning temperature controllers in heat flow–dominated systems. For instance, Karadabağ and Can implemented real-time temperature control of a HFS using a PI-based linear control method^9^. The $\:{K}_{p}$ and $\:{K}_{i}$ parameters of the controller were determined using artificial tree algorithm, particle swarm optimization (PSO), adaptive fire forest optimization, constrained multi-objective state transition algorithm, and differential evolution (DE) optimization algorithms, and the system performance was tested under three different temperature reference values. In^5^, Liu and Pan introduced a PSO-based recurrent closed-loop optimization framework for multiple-controller single-output thermal engineering systems, where controller parameters are tuned sequentially through a recurrent identification–simulation–optimization workflow, leading to significantly improved convergence behavior and practical control performance. Similarly, In^10^, Ekinci proposed a proportional–integral–double-derivative (PIDD^2^) controller optimized by the greater cane rat algorithm for electric furnace temperature regulation, achieving notable reductions in overshoot, settling time, and integral error indices while maintaining robust tracking under disturbances and time-delay conditions. In another study, Ouyang et al. developed an improved sparrow search algorithm incorporating tent mapping initialization, golden-section-based position updating, and Gauss–Cauchy mutation to optimize PID parameters for continuous reactor temperature control, yielding superior convergence speed, global search capability, and robustness compared with classical optimization techniques^11^. Furthermore, recent comparative investigations on temperature control have shown that PID controllers tuned by modern metaheuristic algorithms consistently outperform conventional tuning methods in terms of transient response, steady-state accuracy, and overall objective values, confirming the suitability of metaheuristic-assisted PID frameworks for industrial thermal systems. These advances collectively indicate a clear shift toward optimization-driven temperature control strategies, while also revealing the need for more reliable and computationally efficient optimization schemes tailored to heat flow–dominated processes. Besides optimization-assisted PID tuning approaches, alternative advanced control methodologies have also been investigated for nonlinear dynamic systems. Recently, active disturbance rejection control and model-free control strategies tuned through fictitious reference iterative tuning have demonstrated promising performance in terms of disturbance attenuation, tracking accuracy, and robustness without requiring precise process models^12^. These developments highlight the growing interest in advanced control frameworks capable of maintaining satisfactory performance under modeling uncertainties and external disturbances. Despite these advances, PID-based control architectures remain attractive in industrial applications due to their simplicity, interpretability, ease of implementation, and compatibility with optimization-based tuning procedures.

Beyond direct temperature regulation applications, optimization-based tuning and control strategies have also been extensively investigated in a broad range of thermal, energy, and non-linear dynamic systems, providing important methodological foundations for advanced temperature control design. Wozniak et al. investigated the use of biologically inspired optimization methods for operating a district heating system with minimum cost and maximum efficiency^13^. Polar bear optimization was applied to a mathematical model with two heat exchangers, and the results were compared with the PSO. The findings showed that the proposed method provided effective and efficient results under different weather conditions. Alyoussef et al. proposed novel and robust analytical tuning criteria for PI-PD controllers for unstable and time-delayed processes^14^. To address the time-consuming nature of existing graphical tuning methods and the inadequacy of analytical guidelines, a design approach based on predefined gain and phase margin limits and the center of the stability region has been developed. The proposed method has been tested in real-time on DC-DC buck converters, DC motors, and heat exchangers. The results demonstrated that the method is robust against parameter uncertainties and has provided superior performance with minimal overshoot. Hagglund proposed a novel PID tuning method that is much simpler and faster to implement than current industry standards^15^. The proposed method produces tuning parameters that provide performance close to that of more advanced techniques. The derived method was analyzed to address uncertainties arising from the unknown system model and its intended use, and the results were interpreted accordingly. Peterle et al. have adapted Hagglund’s predictive PID controller, originally developed for first-order systems, to second-order stable systems^16^. Solutions have been developed for real and complex polarity systems; the classic PID structure is preserved, with only an additional linear block added to the system. Thus, while maintaining the basic structure and flexibility of the PID controller, control performance is improved. The proposed structure is called Predictive PID, and simulation results have shown that this method which was able to provide effective and successful performance. Pachauri et al. proposed a novel control approach that combines fractional mathematics with IMC-PID architecture and requires fewer design parameters^17^. First, a fractional-order IMC-PID was designed, and then, to reduce the persistent error, an additional loop with proportional gain was added to develop the MFOIMC-PID structure. The controller parameters were optimized using the Water Cycle Algorithm to obtain the WMFOIMC-PID structure. Simulation results showed that the proposed controller reduced the integral absolute error by 57% and 72%, respectively, compared to FOPID and conventional PID controllers in setpoint tracking. Similar performance improvements were observed in terms of disturbance rejection and noise suppression, thus demonstrating that the WMFOIMC-PID controller is more robust and efficient. In^18^, it was stated that the PID controller tuning for a five-bar planar parallel robot’s highly non-linear trajectory tracking problem has been formulated as an offline non-linear dynamic optimization problem. To ensure stability in the closed-loop system, a dynamic constraint is added to the optimization problem, and eight different variants of the differential evolution algorithm are applied to effectively address this constraint. The comparative results showed that the proposed method achieved faster convergence and reached the appropriate PID parameters. Also, the experimental studies conducted on a real robot prototype have demonstrated the accuracy and practical applicability of the optimization-based tuning approach, as well. Recent advances in intelligent control have also demonstrated the effectiveness of adaptive and learning-based approaches for highly nonlinear systems. For example, a fully actuated system approach-based neuroadaptive controller was developed for underactuated tower crane systems with unavailable states and input dead zones, demonstrating strong robustness and tracking capability under significant nonlinearities and uncertainties^19^. These findings further highlight the growing interest in advanced control frameworks capable of maintaining high performance under complex operating conditions.

Although these studies demonstrate the wide applicability of optimization-based approaches in thermal and non-linear dynamic systems, several limitations remain evident. Most reported temperature regulation strategies rely predominantly on population-based metaheuristic algorithms. While such methods provide global search capability, they often require a high number of function evaluations and may exhibit slow convergence or unstable search behaviour in experimental environments. In addition, the majority of existing works focus on single-degree-of-freedom PID structures, whereas the potential advantages of two-degree-of-freedom PID architectures for improving both reference tracking and disturbance rejection in heat flow systems have not been sufficiently explored. Furthermore, comparatively less attention has been given to lightweight optimization frameworks that can achieve fast convergence with reduced computational complexity while maintaining sufficient local exploitation capability. Quadratic interpolation optimization (QIO) represents one such deterministic approach, where promising regions of the search space are estimated through quadratic model fitting^20^. Recent studies have demonstrated the applicability of QIO and its variants across a wide range of optimization problems. In^21^, binary adaptations of QIO have been successfully developed for solving combinatorial optimization tasks such as the 0–1 knapsack problem, where improved binary QIO formulations have shown superior accuracy and computational efficiency compared to several state-of-the-art metaheuristics. In energy-related applications, QIO has been employed for accurate parameter extraction of photovoltaic cell and module models under varying irradiance conditions, achieving lower modeling errors and faster convergence than conventional optimizers^22^. Moreover, improved quadratic interpolation–based strategies have been integrated into clustering frameworks, such as adaptive K-means algorithms, to enhance centroid initialization and local refinement, yielding better clustering quality on large-scale datasets^23^. QIO has also been utilized in control-oriented applications, including hybrid SA–QIO–tuned PIDF controllers for load frequency control in renewable-integrated multi-area power systems and QIO-optimized observers for sensorless PMSM control, demonstrating strong robustness and superior transient performance^24,25^. Despite these promising results, the conventional formulation of QIO may suffer from stagnation and limited diversity when addressing complex or multimodal optimization landscapes. These observations reveal the need for an optimization framework that enhances convergence reliability without increasing algorithmic complexity, and that can be effectively integrated with an advanced control architecture tailored to practical temperature regulation problems.

In summary, although numerous optimization-based temperature control strategies have been reported in the literature, most existing studies rely on population-based metaheuristic algorithms that often require high computational effort and may suffer from slow convergence or premature stagnation. In addition, the potential advantages of combining lightweight deterministic optimization frameworks with advanced two-degree-of-freedom PID architectures for practical heat-flow systems have not been sufficiently investigated. To address these research gaps, this study proposes an eQIO algorithm and integrates it with a 2DOF-PID controller for real-time temperature regulation. The main contributions of this work are threefold: (i) the development of an enhanced QIO framework incorporating periodic parabolic local refinement and stagnation-aware diversity recovery mechanisms, (ii) a comprehensive benchmark evaluation of the proposed optimizer using the CEC-2019 test suite against several state-of-the-art optimization algorithms, and (iii) the experimental validation of the eQIO-tuned 2DOF-PID controller on a laboratory-scale heat flow system under step, square, and sinusoidal reference signals. The obtained results demonstrate that the proposed framework improves optimization robustness and control performance while preserving the computational simplicity of the original QIO structure.

In this study, an enhanced optimal control framework is proposed for temperature regulation of a laboratory-scale heat flow system. The framework combines a two-degree-of-freedom PID controller with an enhanced quadratic interpolation optimization (eQIO) algorithm developed to address the aforementioned limitations. The enhanced optimizer preserves the original quadratic interpolation structure while incorporating periodic parabolic local refinement to strengthen exploitation and a stagnation detection–based diversity recovery mechanism to prevent premature convergence. The effectiveness of the enhanced optimizer is first validated on the CEC-2019 benchmark suite to evaluate convergence accuracy and statistical reliability. Subsequently, eQIO is employed to tune the two-degree-of-freedom proportional–integral–derivative (2DOF-PID) controller parameters in real-time experimental temperature tracking scenarios involving step, square, and sinusoidal reference signals. The proposed framework aims to achieve improved transient response, reduced steady-state error, lower overshoot, and smoother control effort compared with alternative metaheuristic-based tuning strategies. Through both benchmark evaluation and experimental validation, this study establishes a structured and robust optimization-based tuning methodology for thermal control systems.

## Materıal and methods

This section presents the overall methodology of the study in an integrated manner. First, the experimental heat flow system and its mathematical model are described. Then, the adopted 2DOF-PID control structure for temperature regulation is detailed. Finally, the eQIO optimization algorithm used for tuning the controller parameters and the associated evaluation framework are explained to provide a complete description of the proposed approach and named as eQIO-based 2DOF-PID.

### 2DOF-PID control structure

**Fig. 1:**
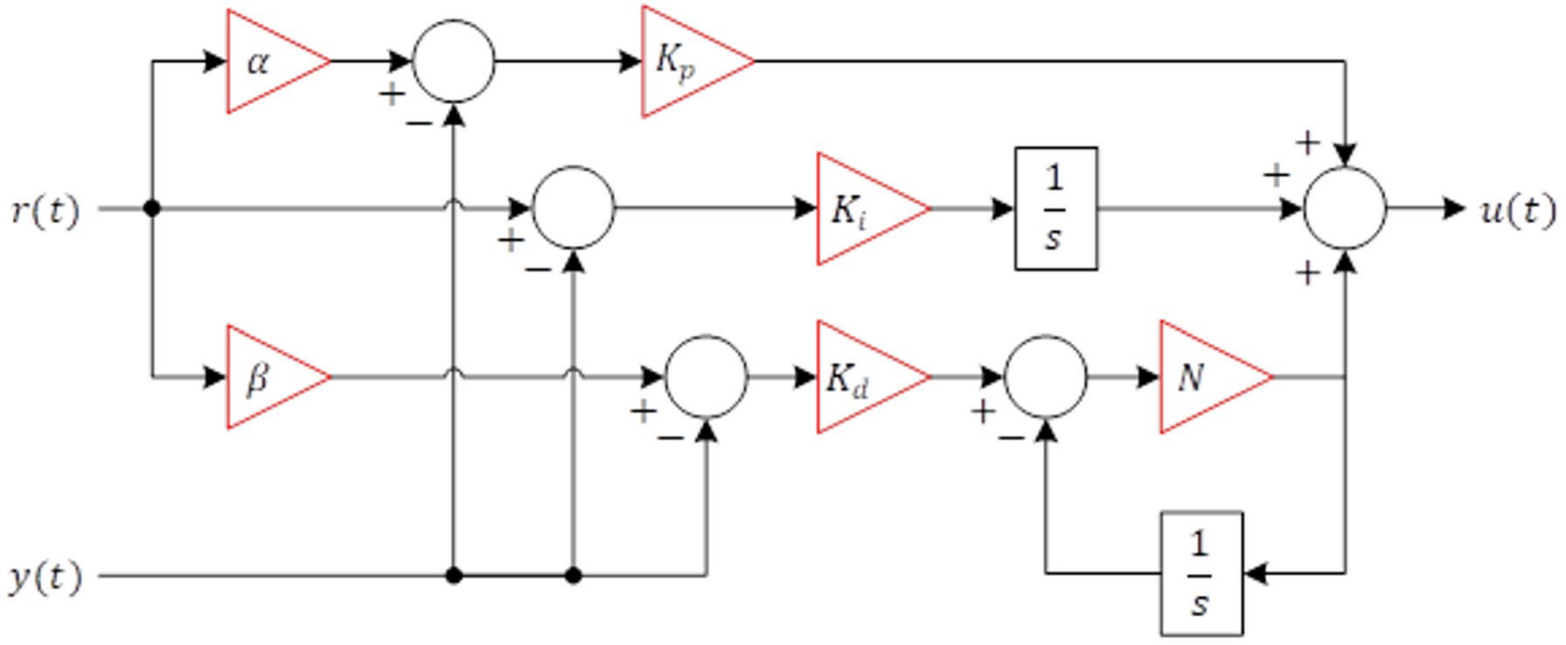
2DOF-PID control structure.

Due to its straightforwardness and robustness, the proportional–integral–derivative (PID) controller remains a widely used controller in industry. In the conventional single-degree-of-freedom (1DOF) PID scheme, a single tracking error signal serves for both reference tracking and disturbance rejection. Although this configuration is admissible in many situations, it results in a fundamental trade-off between transient response and disturbance attenuation. To overcome this limitation, 2DOF-PID control structure shown in Fig. 1 is adopted in this study. The basic concept behind the 2DOF approach is to weight the reference signal differently in the proportional and derivative terms^26–28^. This correction enables shaping the reference response separately from the feedback regulation. In the Laplace domain, the control signal $\:u\left(s\right)$ generated by a 2DOF-PID controller can be expressed as

1 $$\:u\left(s\right)={C}_{r}\left(s\right)r\left(s\right)-{C}_{y}\left(s\right)y\left(s\right)$$

where $\:r\left(s\right)$ denotes the reference input, $\:y\left(s\right)$ is the system output, and $\:{C}_{r}\left(s\right)$ and $\:{C}_{y}\left(s\right)$ represent the reference and feedback components of the controller, respectively.

The controller components are defined as

2 $$\:{C}_{r}\left(s\right)={K}_{p}\alpha\:+\frac{{K}_{i}}{s}+{K}_{d}\beta\:\frac{Ns}{s+N}$$

3 $$\:{C}_{y}\left(s\right)={K}_{p}+\frac{{K}_{i}}{s}+{K}_{d}\frac{Ns}{s+N}$$

Where $\:{K}_{p}$, $\:{K}_{i}$, and $\:{K}_{d}$ are the proportional, integral, and derivative gains,$\:\:\alpha\:$ is the proportional reference weighting factor, $\:\beta\:$ is the derivative reference weighting factor. Substituting (2) and (3) into (1), the explicit control law becomes

4 $$\:u\left(s\right)=\left({K}_{p}\alpha\:+\frac{{K}_{i}}{s}+{K}_{d}\beta\:\frac{Ns}{s+N}\right)\left(r\left(s\right)\right)-\left({K}_{p}+\frac{{K}_{i}}{s}+{K}_{d}\frac{Ns}{s+N}\right)\left(y\right(s\left)\right)$$

5 $$\:u\left(s\right)={K}_{p}\left(\alpha\:r\right(s)-y(s\left)\right)+\frac{{K}_{i}}{s}\left(r\right(s)-y(s\left)\right)+{K}_{d}\frac{Ns}{s+N}\left(\beta\:r\right(s)-y(s\left)\right)$$

In this formulation, the integral term acts on the full tracking error $\:e\left(s\right)=r\left(s\right)-y\left(s\right)$, ensuring zero steady-state error for constant reference inputs. The proportional and derivative terms include reference weighting, which allows independent adjustment of transient tracking behaviour. When connected to a generic plant model $\:G\left(s\right)$, the closed-loop transfer function between the reference input and system output is obtained as

6 $$\:\frac{Y\left(s\right)}{R\left(s\right)}=\frac{G\left(s\right){C}_{r}\left(s\right)}{1+G\left(s\right){C}_{y}\left(s\right)}$$

This expression highlights the fundamental advantage of the 2DOF structure: the numerator, which governs reference tracking, is shaped by $\:{C}_{r}\left(s\right)$, whereas robustness and disturbance rejection characteristics are primarily determined by $\:{C}_{y}\left(s\right)$. The additional tuning parameters $\:b$and $\:c$provide increased flexibility compared to the conventional PID controller. In practice, selecting $\:0\le\:\alpha\:\le\:1$ allows reduction of overshoot during set-point changes, while choosing $\:\beta\:=0$ eliminates derivative action on the reference signal, preventing excessive control activity in response to abrupt reference variations. This general formulation serves as the theoretical basis for the controller implementation described in the subsequent section.

### Heat flow system model

In this study, a laboratory-scale heat flow system (HFS) is utilized as a platform for the investigation of convective heat transfer and closed-loop air temperature control. The experimental setup consists of a resistive heater located at the inlet of an air duct, a blower generating forced airflow, and three temperature sensors positioned at different axial locations along the duct. The objective is to characterize the heat transfer dynamics and regulate the temperature at a selected measurement point. From a thermodynamic perspective, the temperature measured at the $\:n$-th sensor depends on the heater power input, airflow rate, ambient conditions, and sensor position along the duct. Detailed descriptions of the experimental platform can be found in^9,29^.

The mathematical model of the process plant is derived using the thermal energy balance principle. The total thermal energy stored within the system can be expressed as

7 $$\:{E}_{th}=m{C}_{p}T$$

where $\:m$ denotes the equivalent thermal mass of the heated air and surrounding components, $\:{C}_{p}$ is the specific heat capacity, and $\:T$ represents the process temperature. According to the conservation of energy principle, the rate of change of the stored thermal energy is equal to the difference between the supplied thermal power and the heat dissipated to the environment. Therefore,

8 $$\:\frac{d{E}_{th}}{dt}={Q}_{in}-{Q}_{loss}$$

Substituting (7) into (8) yields

9 $$\:m{C}_{p}\frac{dT\left(t\right)}{dt}={Q}_{in}\left(t\right)-{Q}_{loss}\left(t\right)$$

where $\:{Q}_{in}\left(t\right)$ and $\:{Q}_{loss}\left(t\right)$ represent the heat generated by the heater and the heat dissipated to the surroundings, respectively. The heat generated by the electrical heater is assumed to be proportional to the applied control voltage. Accordingly,

10 $$\:{Q}_{in}\left(t\right)={K}_{h}u\left(t\right)$$

where $\:u\left(t\right)$ is the heater input voltage and $\:{K}_{h}$ is the heater gain. The dominant heat dissipation mechanism is convective heat transfer between the heated airflow and the ambient environment. Based on Newton’s law of cooling, the heat loss can be approximated as

11 $$\:{Q}_{loss}\left(t\right)=hA\left[T\left(t\right)-{T}_{amb}\right]$$

where $\:h$ is the effective convective heat transfer coefficient, $\:A$ is the effective heat transfer area, and $\:{T}_{amb}$ denotes the ambient temperature. Substituting (10) and (11) into (9) gives the nonlinear thermal dynamic model.

12 $$\:m{C}_{p}\frac{dT\left(t\right)}{dt}={K}_{h}u\left(t\right)-hA\left[T\left(t\right)-{T}_{amb}\right]$$

For controller design purposes, the system is linearized around a nominal operating point $\:\left({T}_{0},{u}_{0}\right)$. Defining the small-signal variables as

13 $$\:{\Delta\:}T\left(t\right)=T\left(t\right)-{T}_{0}$$

and

14 $$\:{\Delta\:}u\left(t\right)=u\left(t\right)-{u}_{0}$$

the linearized thermal dynamics become

15 $$\:m{C}_{p}\frac{d{\Delta\:}T\left(t\right)}{dt}+hA{\Delta\:}T\left(t\right)={K}_{h}{\Delta\:}u\left(t\right)$$

Applying the Laplace transform under zero initial conditions gives

16 $$\:\left(m{C}_{p}s+hA\right){\Delta\:}T\left(s\right)={K}_{h}{\Delta\:}U\left(s\right)$$

Thus, the transfer function relating the temperature variation to the heater input voltage is obtained as

17 $$\:G\left(s\right)=\frac{{\Delta\:}T\left(s\right)}{{\Delta\:}U\left(s\right)}=\frac{{K}_{h}}{m{C}_{p}s+hA}$$

Defining the steady-state gain and thermal time constant as

18 $$\:K=\frac{{K}_{h}}{hA}$$

and

19 $$\:\tau\:=\frac{m{C}_{p}}{hA}$$

the transfer function can be rewritten in the standard first-order form

20 $$\:G\left(s\right)=\frac{K}{\tau\:s+1}$$

In practical thermal systems, transport delay may arise due to heat propagation and sensor dynamics. Therefore, a first-order plus dead-time (FOPDT) representation can be written as

21 $$\:G\left(s\right)=\frac{K{e}^{-Ls}}{\tau\:s+1}$$

where $\:L$ denotes the equivalent transport delay. Moreover, in this study, the blower speed and sensor location were kept constant during all experiments. Therefore, their effects are embedded in the identified parameters K and τ. Although transport delay may exist in practical thermal systems, system identification results showed that the delay is negligible compared to the dominant thermal time constant. Therefore, based on the experimental system identification, the laboratory-scale HFS employed in this study is represented by the following first-order transfer function

22 $$\:G\left(s\right)=\frac{8}{60s+1}$$

which is adopted throughout the controller design, optimization, and performance evaluation stages.

**Table 1:** The system parameters and physical characteristics of the Heat-Flow System^9,29^.

Symbol	Description	Value	Unit
	HFE dimensions	50 × 15 × 10	cm
	HFE mass	0.5	kg
$\:{V}_{b,nom}$	Blower nominal input voltage	6	V
*B*	Blower nominal airflow	36	CFM
$\:{B}_{SI}$	Blower nominal airflow (in SI units)	1.02	$\:{\mathrm{m}}^{3}/\mathrm{m}\mathrm{i}\mathrm{n}$
$\:{W}_{s}$	Max wind speed	159.4	m/min
$\:{w}_{b,max}$	Blower max speed	2700	RPM
$\:{P}_{h}$	Heater max power (at 5 V)	400	W
$\:{K}_{temp}$	Temperature sensor calibration gain	20	°C$/\mathrm{V}$
*A*	Cross-sectional area	0.0064	$\:{\mathrm{m}}^{2}$
	Current power requirements (maximum current)	5	A
	Heat-flow voltage power requirements	120–240	VAC

The laboratory-scale heat flow system considered in this study can be classified as a stable first-order thermal process with process gain and dominant time constant characteristics. Based on the identified transfer function given in Eq. 22, the process gain and time constant are determined as $\:K=8$ and $\:\tau\:=60$ s, respectively. The plant exhibits stable open-loop behavior since its pole is located at $\:s=-1/60$. Although thermal systems may exhibit transport delays due to heat propagation and sensor dynamics, the delay observed in the employed laboratory setup is negligible compared with the dominant thermal time constant. Therefore, the process is modeled as a first-order process without an explicit dead-time term.

**Fig. 2:**
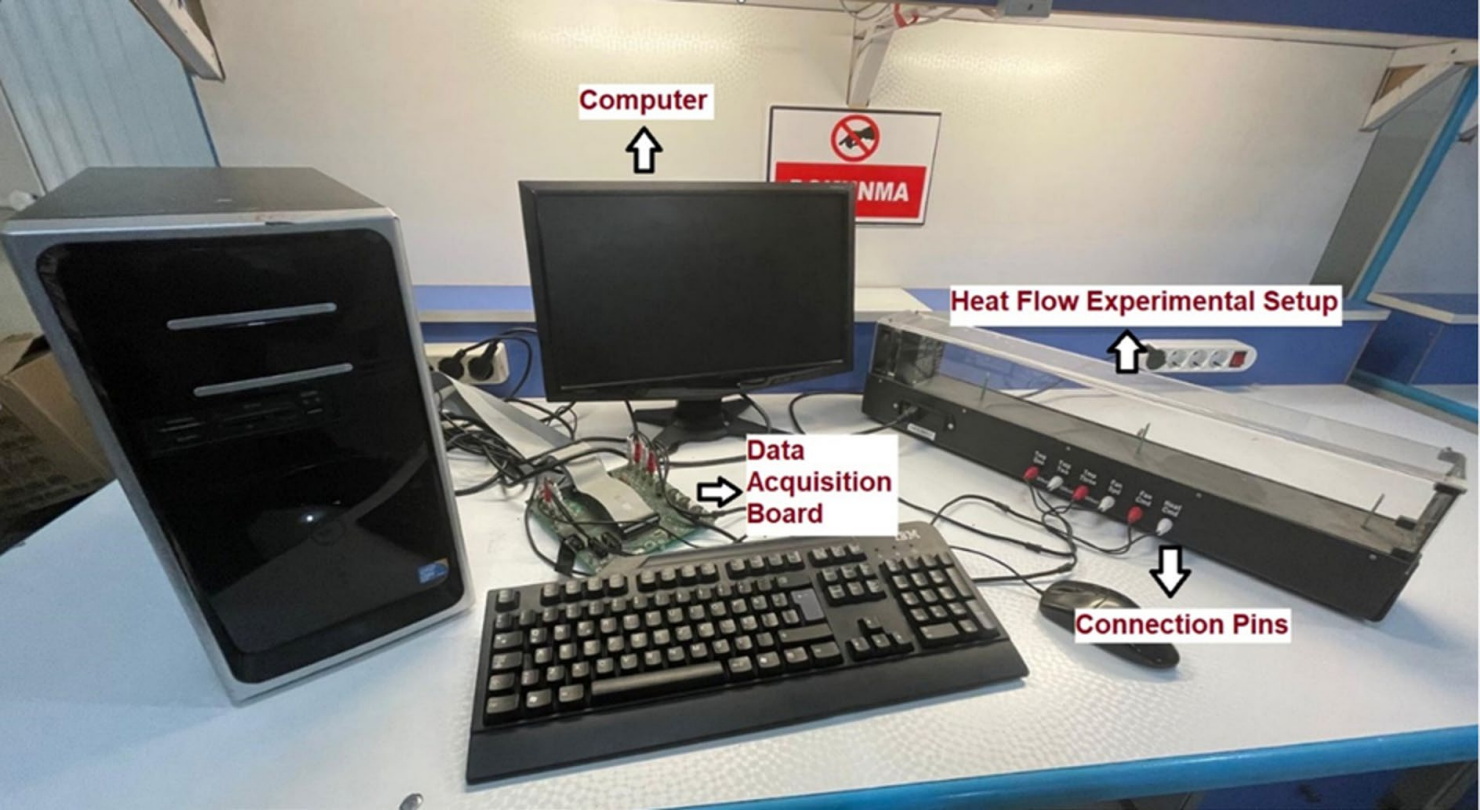
The experimental setup and system components^29^.

A schematic representation of the experimental setup and system components is shown in Fig. 2. The system includes a main control computer, three temperature sensors mounted along the duct, a blower unit supplying airflow, and front-panel connectors providing access to sensor outputs and actuator inputs. Controller tuning and performance analyses are performed using Sensor 1, which is located closest to the heater and serves as the primary feedback variable. The physical characteristics and operating specifications of the HFS are summarized in Table 1.

Before each experimental run, the heat-flow system was allowed to reach thermal equilibrium with the laboratory environment. The initial process temperature was equal to the ambient temperature (approximately 22–25 °C), while the heater input was set to zero. The blower was operated at its nominal speed throughout all experiments to maintain a constant airflow rate. The controller and optimization algorithms were initialized using identical settings for all test scenarios, and data acquisition was started only after the measured temperature signal had stabilized. These conditions ensured repeatability and consistency across all experimental trials.

### Evaluated cost function

In this study, the 2DOF-PID controller parameters are tuned by minimizing a scalar cost function computed from the closed-loop step response. The optimization vector is defined as

23 $$\:\boldsymbol{\theta\:}=[{K}_{p},{\hspace{0.25em}\hspace{0.05em}}{K}_{i},{\hspace{0.25em}\hspace{0.05em}}{K}_{d},{\hspace{0.25em}\hspace{0.05em}}N,{\hspace{0.25em}\hspace{0.05em}}\beta\:,{\hspace{0.25em}\hspace{0.05em}}\alpha\:]$$

where $\:{K}_{p}$, $\:{K}_{i}$, and $\:{K}_{d}$ are the proportional, integral, and derivative gains, respectively; $\:N$ is the derivative filter coefficient; and $\:\alpha\:$ and $\:\beta\:$ are the proportional and derivative set-point weighting factors of the 2DOF-PID controller. For each candidate parameter vector $\:\theta\:$, the closed-loop response of the controlled system is obtained through time-domain simulation under a step reference input $\:r\left(t\right)$ using a fixed sampling interval $\:dt$. The simulated output trajectory is denoted by $\:y\left(t\right)$, and the tracking error is computed as

24 $$\:e\left(t\right)=r\left(t\right)-y\left(t\right)$$

To ensure numerical reliability during the search process, candidate solutions producing undefined trajectories, non-finite values, or invalid simulation outputs are treated as infeasible and assigned a large penalty value $\:J={10}^{8}$. Moreover, standard step-response characteristics are extracted using a 2% settling band. If overshoot or settling time cannot be computed due to an abnormal response, the same penalty is applied. The cost function is constructed by combining normalized tracking accuracy metrics, time-weighted error criteria, transient response measures, steady-state error, and a bound-avoidance barrier term. The tracking accuracy is quantified using the root mean square error (RMSE), and the time-weighted error indices are defined as

25 $$\:\mathrm{R}\mathrm{M}\mathrm{S}\mathrm{E}=\sqrt{\frac{1}{N}\sum\:_{k=1}^{N}{e}^{2}\left({t}_{k}\right)},\:\mathrm{I}\mathrm{T}\mathrm{A}\mathrm{E}={\int\:}_{0}^{{t}_{f}}t\mid\:e\left(t\right)\mid\:dt,\:\mathrm{I}\mathrm{T}\mathrm{S}\mathrm{E}={\int\:}_{0}^{{t}_{f}}t{\hspace{0.17em}}{e}^{2}\left(t\right){\hspace{0.17em}}dt$$

where $\:{t}_{f}$ is the final simulation time and $\:N$ is the number of samples. The steady-state error is computed from the final sample:

26 $$\:{e}_{ss}=\mid\:e\left({t}_{f}\right)\mid\:$$

In addition, the overshoot $\:{M}_{p}$ and the settling time $\:{T}_{s}$ (2% criterion) are computed from the closed-loop response. Since the above metrics have different scales, each term is normalized to yield a dimensionless objective. Let $\:{r}_{\mathrm{m}\mathrm{a}\mathrm{x}}=\mathrm{m}\mathrm{a}\mathrm{x}\mid\:r\left(t\right)\mid\:$. The normalized indices are defined as

27 $$\:{\mathrm{R}\mathrm{M}\mathrm{S}\mathrm{E}}_{n}=\frac{\mathrm{R}\mathrm{M}\mathrm{S}\mathrm{E}}{{r}_{\mathrm{m}\mathrm{a}\mathrm{x}}}$$

28 $$\:{\mathrm{I}\mathrm{T}\mathrm{A}\mathrm{E}}_{n}=\frac{\mathrm{I}\mathrm{T}\mathrm{A}\mathrm{E}}{{t}_{f}^{2}{\hspace{0.17em}}{r}_{\mathrm{m}\mathrm{a}\mathrm{x}}},\:{\mathrm{I}\mathrm{T}\mathrm{S}\mathrm{E}}_{n}=\frac{\mathrm{I}\mathrm{T}\mathrm{S}\mathrm{E}}{{t}_{f}^{2}{\hspace{0.17em}}{r}_{\mathrm{m}\mathrm{a}\mathrm{x}}^{}}$$

29 $$\:{M}_{p,n}=\frac{{M}_{p}}{100},\:,{T}_{s,n}=\mathrm{s}\mathrm{a}\mathrm{t}\left(\frac{{T}_{s}}{{t}_{f}}\right),\:{e}_{ss,n}=\frac{{e}_{ss}}{{r}_{\mathrm{m}\mathrm{a}\mathrm{x}}}$$

where $\:\mathrm{s}\mathrm{a}\mathrm{t}(\cdot\:)$ denotes a saturation operator used to constrain the normalized settling time within a bounded range in case of atypical responses. In addition to performance indices, an interior barrier is introduced to discourage solutions that approach the parameter bounds. Let $\:\mathcal{l}$ and $\:\mathrm{u}$ denote the lower and upper bounds of $\:\theta\:$, respectively, and define $\:\mathrm{s}=\mathrm{u}-\mathcal{l}$. A symmetric barrier term is formulated as

30 $$\:{b}_{i}\left({\theta\:}_{i}\right)=-\mathrm{l}\mathrm{o}\mathrm{g}\left(\frac{\left({\theta\:}_{i}-{\mathcal{l}}_{i}+\epsilon\:\left)\right({u}_{i}-{\theta\:}_{i}+\epsilon\:\right)}{\frac{{s}_{i}^{2}}{4}+\epsilon\:}\right)$$

and the overall barrier penalty is computed by averaging over all parameters:

31 $$\:B\left(\boldsymbol{\theta\:}\right)=\frac{1}{6}\sum\:_{i=1}^{6}{b}_{i}\left({\theta\:}_{i}\right)$$

where $\:\epsilon\:$ is a small constant added to maintain numerical stability. Finally, the scalar objective function minimized in the optimization process is defined as a weighted sum:

32 $$\:J\left(\theta\:\right)={w}_{\mathrm{R}\mathrm{M}\mathrm{S}\mathrm{E}}{\mathrm{R}\mathrm{M}\mathrm{S}\mathrm{E}}_{n}+{w}_{\mathrm{I}\mathrm{T}\mathrm{A}\mathrm{E}}{\mathrm{I}\mathrm{T}\mathrm{A}\mathrm{E}}_{n}+{w}_{\mathrm{I}\mathrm{T}\mathrm{S}\mathrm{E}}{\mathrm{I}\mathrm{T}\mathrm{S}\mathrm{E}}_{n}+{w}_{\mathrm{O}\mathrm{S}}{M}_{p,n}+{w}_{\mathrm{T}\mathrm{s}}{T}_{s,n}+{w}_{\mathrm{e}\mathrm{s}\mathrm{s}}{e}_{ss,n}+{w}_{\mathrm{b}\mathrm{a}\mathrm{r}}B\left(\theta\:\right)$$

Accordingly, the weighting coefficients were determined by considering the relative numerical scales of the normalized performance indices together with the dominant control objectives of the heat-flow system. Since the objective function is a user-defined scalar performance index rather than a physically derived equation, the weighting coefficients were selected empirically through preliminary sensitivity studies and repeated closed-loop simulations. Overshoot and settling time were found to have the strongest influence on thermal safety and operational stability; therefore, higher weights were assigned to these terms. $\:RMSE$ was assigned a relatively high weight to maintain overall tracking accuracy, whereas $\:ITAE$ and $\:ITSE$ were assigned moderate weights to account for accumulated and time-weighted tracking errors. Lower weights were assigned to the steady-state error and barrier terms because their effects are partially reflected by the remaining performance indices and they mainly serve as auxiliary performance and feasibility measures. The final weighting coefficients were normalized such that their sum equals unity, ensuring a balanced aggregation of all performance criteria.

33 $$\:\left[{w}_{\mathrm{R}\mathrm{M}\mathrm{S}\mathrm{E}},{w}_{\mathrm{I}\mathrm{T}\mathrm{A}\mathrm{E}},{w}_{\mathrm{I}\mathrm{T}\mathrm{S}\mathrm{E}},{w}_{\mathrm{O}\mathrm{S}},{w}_{\mathrm{T}\mathrm{s}},{w}_{\mathrm{e}\mathrm{s}\mathrm{s}},{w}_{\mathrm{b}\mathrm{a}\mathrm{r}}]=[0.18,{\hspace{0.25em}\hspace{0.05em}}0.10,{\hspace{0.25em}\hspace{0.05em}}0.15,{\hspace{0.25em}\hspace{0.05em}}0.20,{\hspace{0.25em}\hspace{0.05em}}0.20,{\hspace{0.25em}\hspace{0.05em}}0.10,{\hspace{0.25em}\hspace{0.05em}}0.07\right]$$

### Overview of the proposed eQIO optimization

In this part, we present the eQIO algorithm, which is a variation of the quadratic interpolation optimization method. The eQIO design boils down to keeping the original quadratic interpolation optimization framework and form, designed in such a way that can allow the introduction of separate enhancements to certain points of weakness in the original approach. Therefore, to keep the consistency with the original algorithmic style, the generalized quadratic interpolation rule of exploration–exploitation behavior that determines the exploration, (1), is unchanged for QIO. The proposed alterations are articulated as actions on a conserved nucleus rather than as substitutions of the nucleus. Specifically, eQIO generalizes the original Quadratic Interpolation Optimization (QIO) framework by incorporating two mechanisms: a periodic local refinement strategy based on the best solution and a stagnation-aware diversity recovery strategy to manage population dynamics through the search process. These modifications are introduced in such a way that they do not change the original interpolation equations, but they can be considered as corrective terms, thereby implying improved stability and robustness of the resulting convergence. For the sake of simplicity, the initialization procedure and the original QIO-based generalized quadratic interpolation search method will first be explained in this section. Next, the newly introduced enhancement module is elaborated and the complete eQIO algorithmic flow is described. This well-structured presentation clearly separates the contributions of the proposed method from what is kept in the original QIO scheme^20^.

#### Initialization phase

At the beginning of the search process, a population of $\:N$ candidate solutions is randomly generated within the predefined search bounds. Each candidate represents a solution vector in a $\:D$-dimensional decision space defined as

34 $$\:\mathbf{x}=[{x}_{1},{x}_{2},\dots\:,{x}_{D}]\in\:{\mathbb{R}}^{D}$$

Each decision variable $\:{x}_{j}$ is bounded within predefined lower and upper limits, denoted by $\:{l}_{j}$ and $\:{u}_{j}$, respectively. The initial position of the $\:i$-th individual in the $\:j$-th dimension is given by

35 $$\:{x}_{i,j}={l}_{j}+{r}_{i,j}{\hspace{0.17em}}({u}_{j}-{l}_{j}),$$

where $\:{r}_{i,j}\sim\:\mathcal{U}\left(\mathrm{0,1}\right)$ is a uniformly distributed random number. This initialization strategy ensures a broad and unbiased coverage of the feasible domain, providing sufficient diversity for the subsequent search process. After population initialization, the objective function values of all individuals are evaluated:

36 $$\:{f}_{i}=f\left({\mathrm{x}}_{i}\right),i=\mathrm{1,2},\dots\:,N.$$

where $\:f\left(\mathrm{x}\right)\:$denotes the objective (fitness) function evaluated at the candidate solution vector $\:\mathrm{x}$. The optimization process aims to minimize $\:f\left(\mathrm{x}\right)$, and lower values indicate better solution quality. The best solution in the initial population is then identified as

37 $$\:{\mathrm{x}}^{best}=\mathrm{a}\mathrm{r}\mathrm{g}\underset{{\mathrm{x}}_{i}}{\mathrm{m}\mathrm{i}\mathrm{n}}{f}_{i},$$

with the corresponding best objective value denoted by $\:{f}^{best}=f\left({\mathrm{x}}^{best}\right)$. This initial best solution serves as a reference point for the exploitation-oriented update rules and enhancement mechanisms introduced in the later stages of the algorithm. The initialization phase is identical to that of the original QIO framework and does not introduce any additional parameters or modifications^20^.

#### Generalized quadratic ınterpolation mechanism

The search mechanism of quadratic interpolation optimization is based on the analytic minimization of a second-order interpolation function constructed from multiple candidate solutions. This mechanism, referred to as generalized quadratic interpolation (GQI), constitutes the core of the QIO algorithm and is fully preserved in the proposed eQIO framework. For a given dimension $\:j$, consider three scalar points $\:{x}_{i,j}$, $\:{x}_{{k}_{1},j}$, and $\:{x}_{{k}_{2},j}$, with corresponding objective function values $\:{f}_{i}=f\left({\mathbf{x}}_{i}\right)$, $\:{f}_{{k}_{1}}=f\left({\mathbf{x}}_{{k}_{1}}\right)$, and $\:{f}_{{k}_{2}}=f\left({\mathbf{x}}_{{k}_{2}}\right)$. Using these three points, a quadratic interpolation polynomial is implicitly defined, and its minimizer can be analytically obtained as

38 $$\:{x}_{j}^{\mathrm{*}}=\frac{({x}_{{k}_{1},j}^{2}-{x}_{{k}_{2},j}^{2}){f}_{i}+({x}_{{k}_{2},j}^{2}-{x}_{i,j}^{2}){f}_{{k}_{1}}+({x}_{i,j}^{2}-{x}_{{k}_{1},j}^{2}){f}_{{k}_{2}}}{2\left[({x}_{{k}_{1},j}-{x}_{{k}_{2},j}){f}_{i}+({x}_{{k}_{2},j}-{x}_{i,j}){f}_{{k}_{1}}+({x}_{i,j}-{x}_{{k}_{1},j}){f}_{{k}_{2}}\right]}.$$

Since the relative ordering of the three points may vary, the QIO framework employs a guarded formulation of the interpolation operator. In this formulation, the scalar values $\:{x}_{i,j}$, $\:{x}_{{k}_{1},j}$, and $\:{x}_{{k}_{2},j}$ are reordered according to their corresponding objective function values, and reflection rules are applied when necessary to ensure that the computed minimizer remains within a valid search interval. This guarded interpolation operator is denoted as

39 $$\:{x}_{j}^{\mathrm{*}}=\mathrm{G}\mathrm{Q}\mathrm{I}({x}_{i,j},{x}_{{k}_{1},j},{x}_{{k}_{2},j}),$$

which guarantees a feasible and numerically stable update for all possible configurations. During the iterative search process, each individual is updated using either an exploration-oriented or exploitation-oriented strategy. In the exploration phase, the current individual and two randomly selected individuals from the population are used to generate a new candidate position as

40 $$\:{x}_{i,j}^{new}=\mathrm{G}\mathrm{Q}\mathrm{I}({x}_{i,j},{x}_{{k}_{1},j},{x}_{{k}_{2},j}),{k}_{1}\ne\:{k}_{2}\ne\:i.$$

A further perturbation of this update is a stochastic term, which diminishes in magnitude with iteration count, allowing for an initial wide exploration of the state space and a reduced amount of randomness as the algorithm converges. During the exploitation phase, the global best solution $\:{\mathbf{x}}^{best}\:$is directly used in the interpolation to focus the search in the vicinity of promising areas. The corresponding update rule can be written as

41 $$\:{x}_{i,j}^{new}=\mathrm{G}\mathrm{Q}\mathrm{I}({x}_{{k}_{1},j},{x}_{{k}_{2},j},{x}_{j}^{best}).$$

Boundary violations are corrected by re-sampling infeasible components uniformly within their permitted domains after every update. The new candidate solution becomes the current one if, and only if, it leads to an improved value of the objective function, resulting in a greedy selection rule. Note that the generalized quadratic interpolation relations and the update schemes given in this subsection are employed as they are in the proposed eQIO algorithm. The improvements that will be introduced in the subsequent subsections add on top of this classic QIO core and do not interfere with the underlying mathematical essence of the algorithm.

#### Periodic parabolic local search

While the generalized quadratic interpolation approach of QIO gives a descent direction that makes efficient use of second-order information, its update rule is by nature single-step and population-based. Consequently, when the population converges around a promising region, progress may decelerate due to the absence of an explicit local refinement mechanism. To overcome this limitation, eQIO employs a periodic parabolic local search approach on the current best solution.

Let $\:{\mathrm{x}}^{best}=[{x}_{1}^{best},{x}_{2}^{best},\dots\:,{x}_{D}^{best}]$denote the best solution obtained at iteration $\:t$. At predefined iteration intervals, a one-dimensional quadratic model is constructed independently for each decision variable $\:{x}_{j}$in the neighborhood of $\:{x}_{j}^{best}$. Three sampling points are defined as

42 $$\:{x}_{j}^{\left(-\right)}={x}_{j}^{best}-{r}_{j},{x}_{j}^{\left(0\right)}={x}_{j}^{best},{x}_{j}^{\left(+\right)}={x}_{j}^{best}+{r}_{j},$$

where $\:{r}_{j}$ represents the local search radius for the $\:{j}_{th}$ dimension. The corresponding objective function values

43 $$\:{f}^{\left(-\right)}=f\left({\mathbf{x}}^{\left(-\right)}\right),{f}^{\left(0\right)}=f\left({\mathbf{x}}^{best}\right),{f}^{\left(+\right)}=f\left({\mathbf{x}}^{\left(+\right)}\right)$$

are used to fit a quadratic function in the normalized coordinate system. The minimizer of this quadratic approximation is analytically obtained as

44 $$\:{t}_{j}^{\mathrm{*}}=\frac{{f}^{\left(-\right)}-{f}^{\left(+\right)}}{2\left({f}^{\left(-\right)}-2{f}^{\left(0\right)}+{f}^{\left(+\right)}\right)}.$$

The refined solution for the $\:j$-th dimension is then computed as

45 $$\:{x}_{j}^{new}={x}_{j}^{best}+{t}_{j}^{\mathrm{*}}{\hspace{0.17em}}{r}_{j}.$$

For numerical stability and to avoid too large displacements, the value of $\:{t}_{j}^{\mathrm{*}}\:$is restricted to a certain predefined interval. Any violation of boundaries that might be produced by this update is corrected by projecting the solution onto the feasible search space. The local search radius $\:{r}_{j}\:$is adaptively decreased at the iterations according to

46 $$\:{r}_{j}=\alpha\:\left(t\right){\hspace{0.17em}}({u}_{j}-{l}_{j}),$$

where $\:\alpha\:\left(t\right)$ is a monotonically decreasing function of the iteration number. This adaptive strategy allows for more local exploration at the beginning of the optimization procedure and more accurate local refinement toward the end of convergence. Upon the completion of the parabolic refinement in each dimension, the locally enhanced solution replaces the current best solution if it has a lower objective function value. By integrating this periodic parabolic local search into the main optimization loop, eQIO strengthens exploitation capability without modifying the original QIO interpolation equations or adding new control parameters into the population update rules.

#### Stagnation detection and diversity recovery

While the periodic parabolic local search improves exploitation capability around the best solution, population-based optimization algorithms may still experience stagnation when successive iterations fail to produce meaningful improvements. In the original QIO framework, no explicit mechanism exists to detect or mitigate such stagnation, which may lead to prolonged search inactivity around suboptimal regions. To overcome this limitation, eQIO incorporates a stagnation detection and diversity recovery strategy as a second enhancement.

Let $\:{f}^{best}\left(t\right)$denote the best objective function value obtained at iteration $\:t$. The search process is considered stagnated if the improvement in the best fitness value remains below a small threshold $\:\epsilon\:$over consecutive iterations, which can be expressed as

47 $$\:\mid\:{f}^{best}\left(t\right)-{f}^{best}(t-1)\mid\:<\epsilon\:.$$

If this condition persists for a predefined number of iterations, the algorithm assumes that the population has converged prematurely or entered a flat region of the search space. Once stagnation is detected, eQIO activates a controlled diversity recovery mechanism. Instead of fully reinitializing the population, a fraction of the worst-performing individuals is selected based on their objective function values. Let $\:\mathcal{W}$denote the index set of these individuals. Each selected individual is then relocated in the neighborhood of the current best solution according to

48 $$\:{\mathrm{x}}_{i}^{new}={\mathrm{x}}^{best}+{\eta\:}_{i}\odot\:\sigma\:\left(t\right),i\in\:\mathcal{W},$$

where $\:{\eta\:}_{i}\sim\:\mathcal{N}(0,\mathrm{I})$ is a Gaussian random vector and $\:\sigma\:\left(t\right)$ is an iteration-dependent scaling vector. The scaling term $\:\sigma\:\left(t\right)$ is decreased with the increase in iteration, so that early diversity recovery can guarantee global exploration around the best solution, whereas later diversity recovery performs fine-tuning of the solutions. The displaced solutions are projected back into the admissible search space when boundary violations occur.

Redistributing only the weakest individuals and anchoring the recovery around the best solution enable eQIO to retain accumulated search knowledge and impart controlled diversity to the population. This results in more efficient prevention of long stagnation, while not affecting the convergence property driven by the generalized quadratic interpolation and local refinement phases. The two improvement components described above are motivated by the same idea and combined as lightweight modules operating on top of QIO as the basic principle. More specifically, the periodic parabolic refinement is performed at fixed intervals to refine the best solution, while the stagnation-aware diversity recovery is activated only when there is no best-so-far improvement for a certain number of iterations. For the sake of clarity, the entire procedure of the proposed eQIO algorithm is summarized as follows.

### Justification of eQIO selection, optimization settings, and statistical evaluation

The modification is intended to enhance the convergence reliability and robustness of the original QIO without changing its intrinsic search nature and compared with the mountain gazelle optimization (MGO)^30^, tuned moss growth optimization (TMGO)^31^, whale optimization algorithm (WOA)^32^, and superb fairy-wren optimization algorithm (SFOA)^33^ algorithms. To guarantee a fair and unbiased comparison, all algorithms use the same population size, $\:N=30$, and the same number of iterations, $\:T=100$, resulting in an equal total number of objective function evaluations of 3000. The main internal hyperparameters of each algorithm are shown in Table 2.

**Table 2:** Optimization settings for all compared algorithms.

Algorithm	Pop.	Iter.	Total Eval.	Main Internal Hyperparameters
eQIO	30	100	3000	$\:eps\:=\:1e-12$; $\:{w}_{1}=\:0.3$; $\:{w}_{2}=\:0.3$; $\:worst\_replace\_frac\:=\:0.25$; $\:{alpha}_{0}\:=\:0.25$; $\:{alpha}_{T}\:=\:0.05$; $\:{alpha\_div}_{0}=\:0.5$; $\:a{lpha\_div}_{T}=\:0.1$
QIO				$\:eps\:=\:1e-12;{w}_{1}\:=\:3.0;\:{w}_{2}\:=\:3.0$
MGO				$\:alpha\:=\:0.5$; $\:beta\:=\:0.5$; $\:delta\:=\:0.3$; $\:{w}_{start\:}=\:0.9$; $\:{w}_{drop\:}=\:0.5$; $\:{k}_{frac\:}=\:0.125$
TMGO				w = 2; $\:{\mathrm{r}\mathrm{e}\mathrm{c}}_{num}=\:10$; $\:{\mathrm{d}}_{1}\:=\:0.2$;
SFOA				$\:eps\:=\:1e-12$; $\:{levy}_{beta}=\:1.5$; $\:{levy}_{scale}=\:0.5$; $\:{k}_{scale}\:=\:0.2$; $\:C\:=\:1$; $\:{s}_{scale}\:=\:20.0$; $\:T\:=\:0.5$; $\:{m}_{scale}\:=\:2.0$; $\:{p}_{{scale}_{1}}\:=\:2.0$; $\:{p}_{{scale}_{2}}\:=\:1.0$
WOA				$\:b\:=\:1.0$; $\:{a}_{start\:}=\:2.0$; $\:{a}_{end\:}=\:0.0$; $\:{a}_{{2}_{start}}=\:-1.0$; $\:{a}_{{2}_{end}}\:=\:-2.0$; $\:{p}_{switch\:}=\:0.5$; $\:{c}_{scale}\:=\:2.0$; $\:{a}_{scale}\:=\:2.0$;

The parameter settings for the competing approaches were adopted from their original studies, whereas the hyperparameters of eQIO were determined empirically through preliminary experiments to provide a suitable balance between exploration capability, exploitation strength, convergence stability, and computational cost. In particular, the local refinement and diversity recovery parameters were selected to improve convergence reliability while preserving the lightweight structure of the original QIO framework. Larger values of the local refinement parameters increased exploitation capability and accelerated local convergence, whereas excessively small values reduced the effectiveness of the refinement stage. Similarly, higher diversity recovery settings improved population diversity and reduced the risk of premature convergence, while overly aggressive replacement strategies increased randomness and slowed convergence. The adopted parameter values provided the most stable convergence behavior and the best overall optimization performance during preliminary experiments. The same hyperparameter settings were maintained throughout all benchmark and controller optimization studies to ensure a fair and reproducible comparison. All algorithms were run independently 500 times due to their stochastic nature. The quality of the solutions, convergence consistency, and robustness of the algorithms were assessed using best, mean, and standard deviation statistics.

## Results and dıscussıons

In this section, eQIO is tested on a number of classical benchmark functions to compare its search ability, robustness, and convergence with other state-of-the-art metaheuristic algorithms. Then, the superiority of the eQIO-based 2DOF-PID controller is demonstrated through a case study on the optimal control of the heat flow system, where the optimization performance and control response characteristics are studied. The results are interpreted to highlight the strengths and weaknesses of the techniques described herein.

### Benchmark functions

In order to show its effectiveness, the eQIO algorithm is tested on ten benchmark functions from the CEC-2019 test suite^34^, which is the standard test suite for metaheuristic optimization algorithms. This benchmark provides an unobstructed view of convergence behavior, local optima resistance, exploration–exploitation trade-off, and scalability. The adopted test functions are as in Table 3, and they have been selected such that they are multimodal, non-separable, oscillatory, and have complex dependencies among variables. These deviations permit the study of the algorithm’s performance in different mathematical and structural scenarios.

**Table 3:** CEC2019 Test Functions^34^.

No	Function	D	Search Range	Best
$\:{\mathcal{F}}_{1}$	Storn’s Chebyshev Polynomial Fitting Problem	9	[–8192, 8192]	1
$\:{\mathcal{F}}_{2}$	Inverse Hilbert Matrix Problem	16	[− 16,384, 16,384]	1
$\:{\mathcal{F}}_{3}$	Lennard–Jones Minimum Energy Cluster	18	[− 4, 4]	1
$\:{\mathcal{F}}_{4}$	Rastrigin’s Function	10	[− 100, 100]	1
$\:{\mathcal{F}}_{5}$	Griewangk’s Function	10	[− 100, 100]	1
$\:{\mathcal{F}}_{6}$	Weierstrass Function	10	[− 100, 100]	1
$\:{\mathcal{F}}_{7}$	Modified Schwefel’s Function	10	[− 100, 100]	1
$\:{\mathcal{F}}_{8}$	Expanded Schaffer’s F6 Function	10	[− 100, 100]	1
$\:{\mathcal{F}}_{9}$	Happy Cat Function	10	[− 100, 100]	1
$\:{\mathcal{F}}_{10}$	Ackley Function	10	[− 100, 100]	1

Overall, these benchmark problems provide a reliable and standardized basis for evaluating the convergence accuracy and robustness of the proposed method across diverse optimization landscapes.

### Proposed eQIO performance comparison

**Fig. 3:**
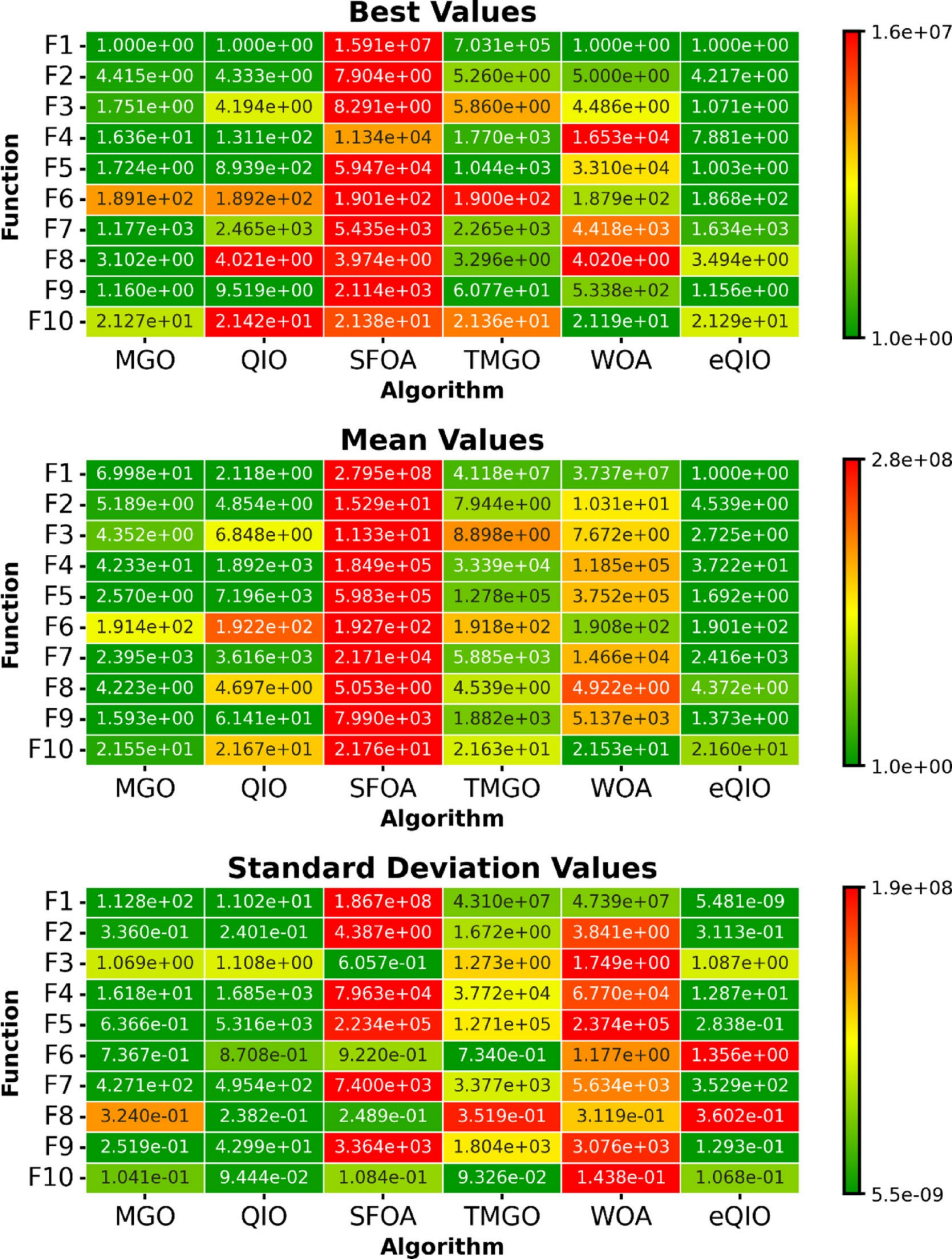
CEC 2019 comparison of the optimization algorithms with heatmap.

Following standard practice, the performance of eQIO relative to competing methods is reported in terms of the best, mean, and standard deviation from 30 independent runs on each benchmark function as shown in Fig. 3. In short, eQIO significantly outperforms the baseline QIO in solution quality and reliability, and the improvement also achieves competitive results with other metaheuristic optimizers. In particular, it is clear that eQIO strongly acquires convergent stability for the numerically sensitive, structured problems F1–F3. eQIO always finds the global optimum on F1, a unit mean value, and a very small standard deviation, whereas QIO attains a far larger mean value. Although MGO is also able to reach small mean values for this function, the variance of eQIO is much lower, suggesting that the results among the various runs are more consistent. The same pattern can be seen in F2, for which all algorithms return more or less similar best and average values, but eQIO obtains the smallest mean error together with a small standard deviation, outperforming QIO slightly and being comparable to MGO and TMGO. These results demonstrate that the proposed improvements do not jeopardize the numerical stability on ill-conditioned problems. On F3, modeling a more complex energy minimization, eQIO again achieves better average performance and smaller variance than QIO, suggesting better robustness to highly sensitive search landscapes.

F4–F7 are strongly multimodal and deceptive functions, and the optimal solution is usually difficult to be approached prematurely for these problems. Accordingly, eQIO again has a large advantage over QIO, in particular in terms of average performance on F7. QIO and SFOA have the worst performance, with large mean error values and variances, whereas eQIO obtains smaller mean values with also much smaller variances, signifying its power in tackling landscapes with an extremely large number of local optima. A great improvement can be found on F5, where eQIO finds near-optimal best values and the smallest mean error when compared to other competitors, and TMGO, QIO, WOA, and SFOA mean and standard deviation values are much higher. This behavior states that the new operators can achieve stronger exploitation ability. For F6, the best values of all algorithms are in the same magnitude; nevertheless, the smallest mean value belongs to eQIO among all the competing approaches, implying that eQIO exhibits a more consistent convergence trend even in complex search spaces, although it is not guaranteed to yield the smallest standard deviation. For F7, eQIO demonstrates a remarkable enhancement over the vanilla QIO in both best and mean results. Although MGO attains the minimum mean value for this function, eQIO performs better than TMGO, WOA, and SFOA and has smaller variability than QIO, which again confirms its better robustness.

For the two non-separable functions F8 and F9, which involve strong interactions among decision variables, the eQIO method attains near-best values on both and also produces low mean errors with small standard deviations. In addition, it should be noted that, while QIO and SFOA exhibit larger dispersion, eQIO converges more robustly, indicating that eQIO has better coordination among decision variables when dealing with complex non-separable search spaces. Finally, for F10, the best and mean values produced by all algorithms are very close to each other, which is consistent with the relatively smooth nature of the function. In this case, eQIO exhibits performance comparable to that of the other methods, without introducing additional variance in the results, once again confirming that the proposed improvements do not compromise performance on simpler landscapes. In conclusion, the results show that eQIO outperforms its baseline QIO on average in terms of both the quality of the solution and the stability for most of the benchmark functions. Although eQIO is not the best for every single problem, it provides a good balance between exploration and exploitation and converges robustly to different test and real-world optimization problems.

**Fig. 4:**
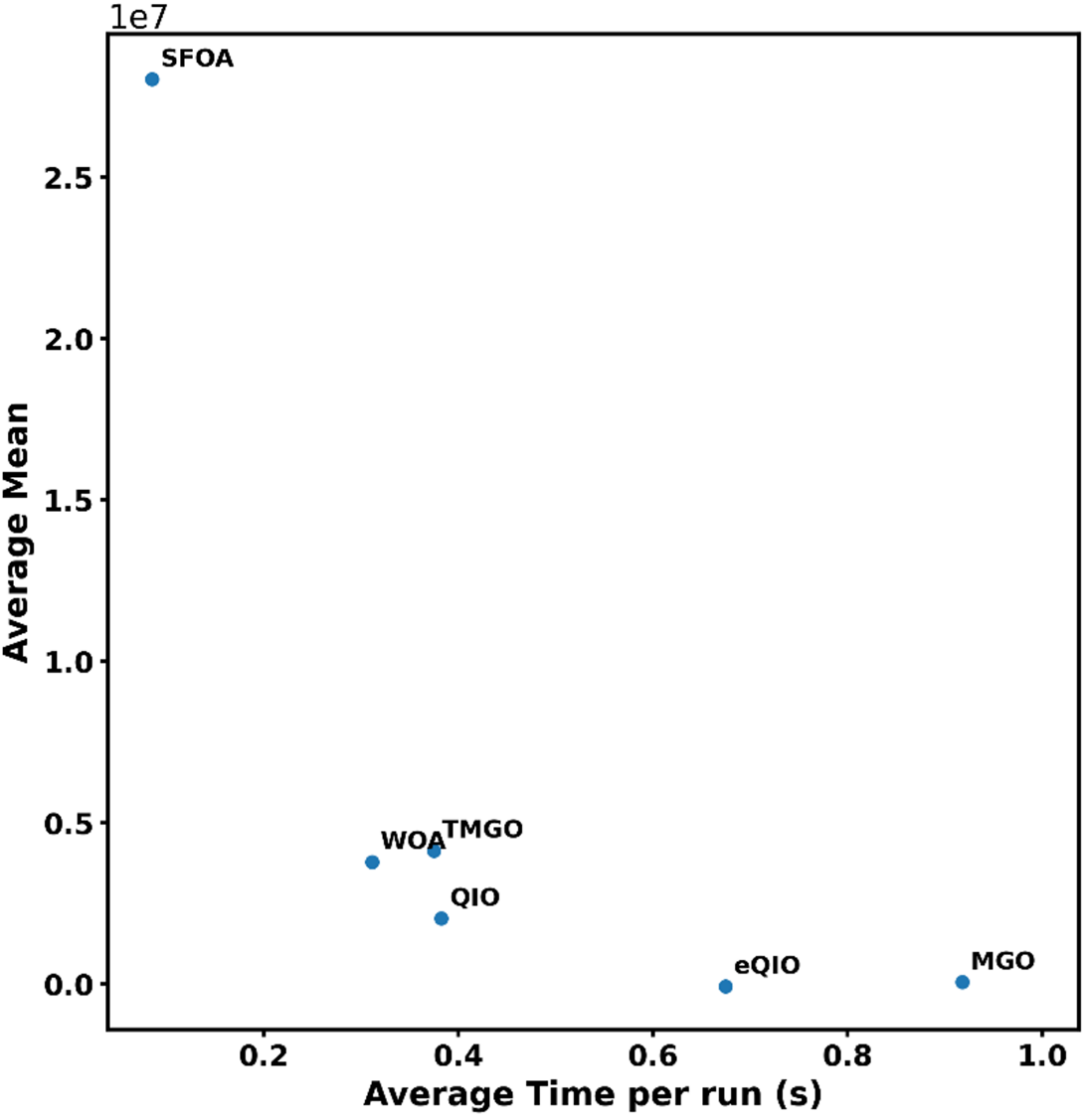
Comparative runtime–performance analysis of the algorithms.

Figure 4 compares the complexity (average runtime) with the accuracy (average objective value) of the algorithms. From the figure, one can observe that eQIO lies in the desirable region of the plot and achieves low objective function values with a modest amount of computational time. This indicates that eQIO achieves a good trade-off between solution quality and runtime. Although MGO can obtain comparable or slightly better average objective values, it requires the longest runtime among all methods. On the contrary, the fastest algorithm, SFOA, provides the worst average objective values, which means its exploitation ability is poor. QIO, TMGO, and WOA can be seen to lie somewhere in between, but still do not offer the same combination of low average error and reasonable runtime as eQIO.

**Fig. 5:**
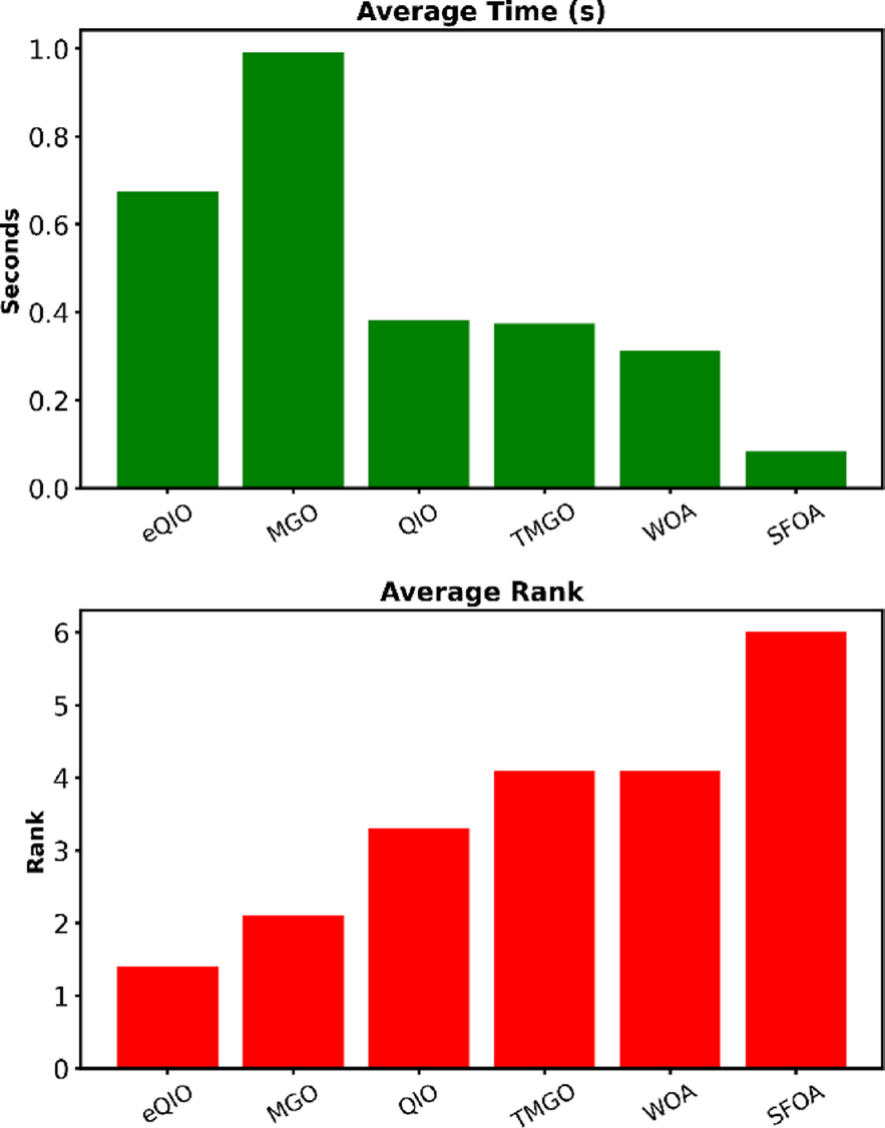
Computational time and average rank comparison of the algorithms.

In Fig. 5, the mean rank and the mean runtime of each algorithm across all benchmark functions are reported. SFOA is the fastest method in terms of CPU time, whereas MGO is the slowest. The runtime of eQIO is longer than that of the baseline QIO due to the inclusion of local search and stagnation-handling mechanisms, but the overhead remains limited. More importantly, eQIO obtains the best average rank, which means that eQIO achieves the best overall performance on the entire benchmark set. The next best performers are MGO and QIO in the second and third positions, while TMGO and WOA are tied for fourth and fifth place. SFOA is consistently the weakest performer.

**Fig. 6:**
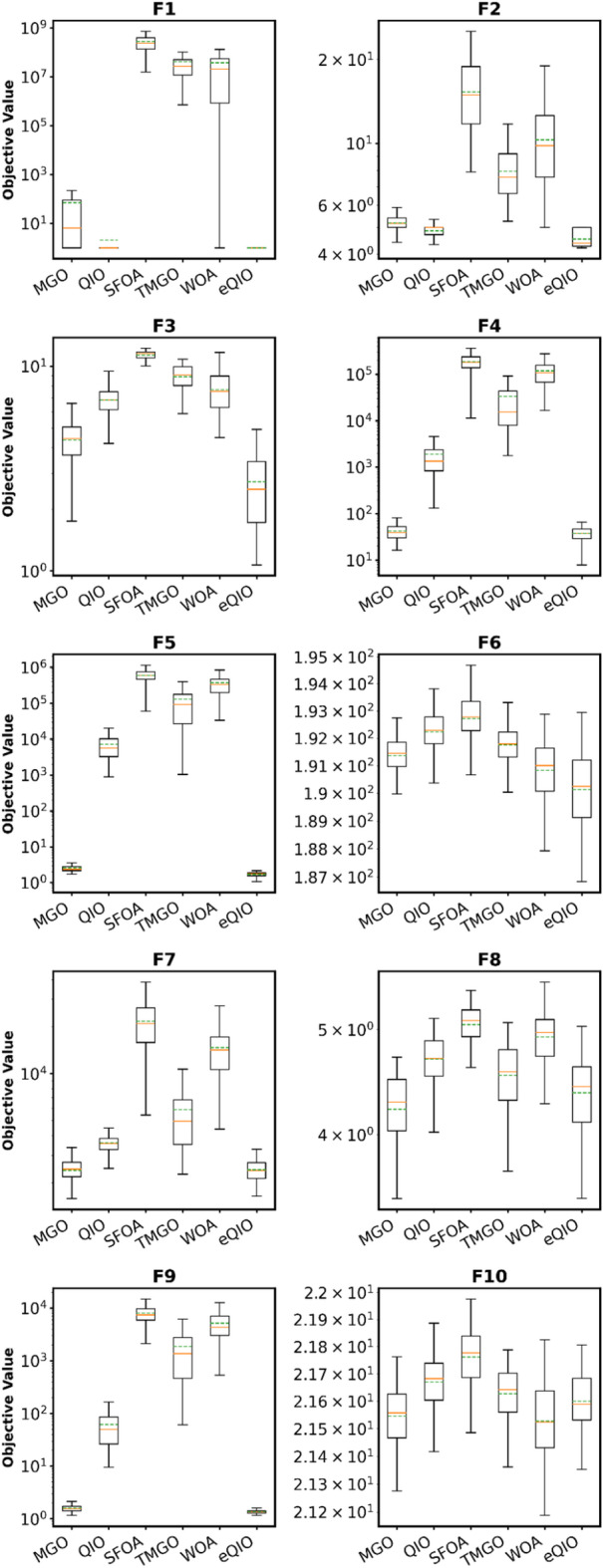
Boxplot analysis of the algorithms.

The boxplots of the objective values of functions F1–F10 are presented in Fig. 6, where both precision and robustness can be observed. For both F1 and F5, eQIO obtains a small interquartile range and a low median, which implies that the convergence is stable and good near-global optimum solutions are obtained. For F2 and F3, eQIO shows a much smaller variance than both QIO and SFOA, which demonstrates that the developed enhancements significantly improved run-to-run consistency. Conversely, for the highly multimodal F4 and F7 problems, eQIO attains median values that are significantly lower than those obtained by TMGO, WOA, and SFOA, further validating its enhanced capability to escape local optimal solutions. For F6, the distributions of the results are very tightly clustered among all the methods, but eQIO yields a slightly smaller median and variance. The functions F8–F10 are fairly mild, and eQIO still produces competitive medians and reasonably low variances, indicating that the enhancements do not cause a performance loss, even for less complex landscapes.

### 2DOF-PID parameter tuning with optimization

**Fig. 7:**
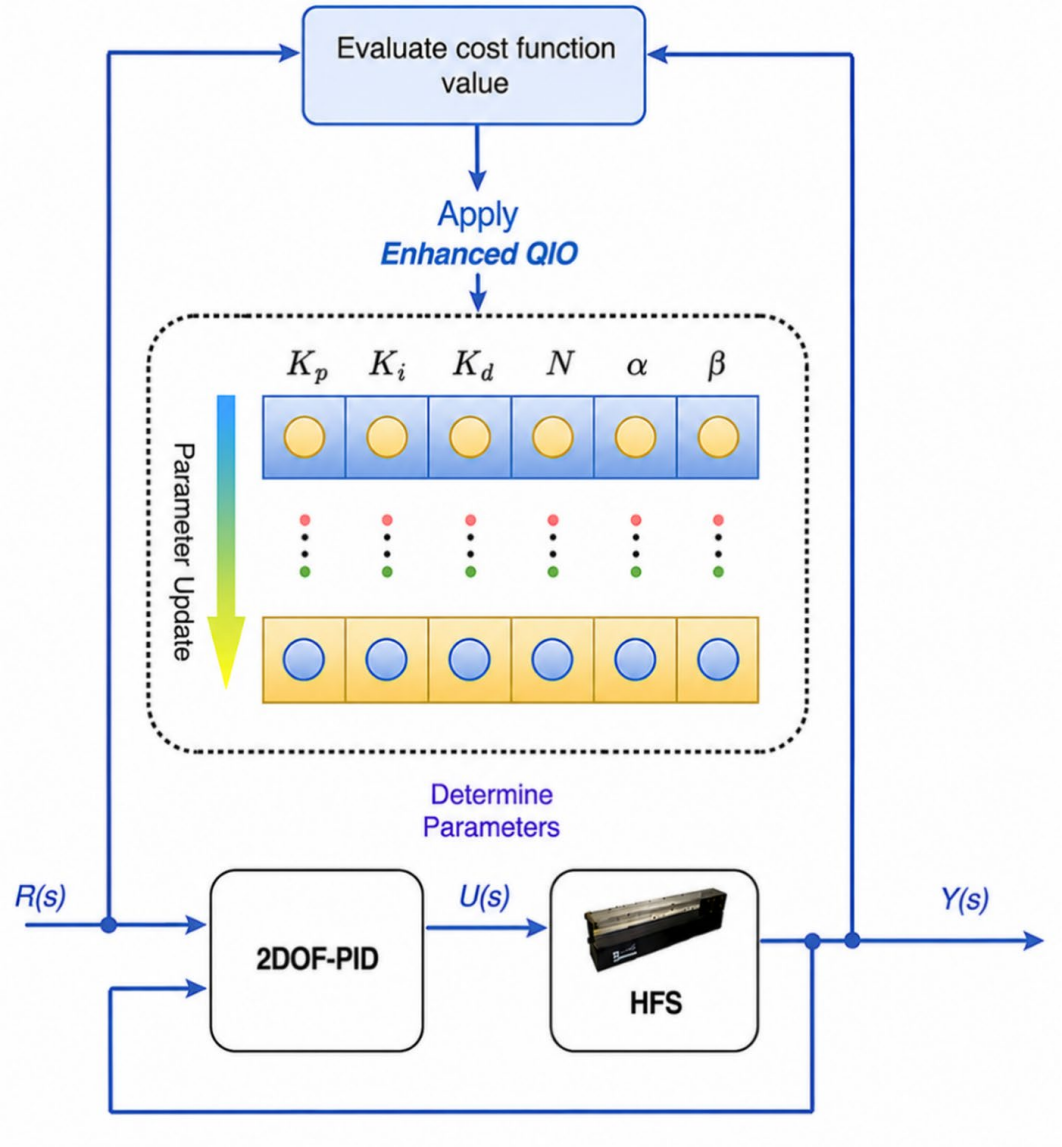
Block diagram of eQIO-based 2DOF-PID control system for HFS.

In this study, the parameters of the proposed 2DOF-PID controller were optimized using six different metaheuristic algorithms: QIO, eQIO, MGO, TMGO, WOA, and SFOA. The optimization process aimed to determine the optimal set of controller parameters $\:{K}_{p}$, $\:{K}_{i}$, $\:{K}_{d}$, $\:\mathrm{N}$, $\:\beta\:$, and $\:\alpha\:$ that minimize the predefined performance index described in Sect.  2.3. The same search bounds, population size, and evaluation budget were used for all algorithms to ensure a fair comparison. The general structure of the proposed algorithm and the control system is given in Fig. 7.

The controller parameters were randomly initialized within predefined search intervals, namely $\:{K}_{p}\in\:[1,\:4]$, $\:{K}_{i}\in\:[0,\:0.5]$, $\:{K}_{d}\in\:[0,\:0.1]$, $\:N\in\:[0.5,\:3]$, $\:\alpha\:\in\:[1,\:1.3]$, and $\:\beta\:\in\:[0.8,\:1.0]$. These bounds were selected based on preliminary experiments and practical operating requirements of the heat-flow system to ensure feasible and stable controller configurations throughout the optimization process. The final values of the parameters found by each method are listed in Table 4.

Overall, all considered algorithms are capable of tuning the 2DOF-PID controller to satisfactory parameter regions. The parameters obtained by eQIO exhibit a relatively balanced distribution among the proportional, integral, and derivative terms. In conjunction with the statistical performance results presented in the preceding subsection, these findings suggest that the proposed QIO-based mechanism provides a robust and reliable parameter tuning capability for delayed 2DOF-PID control systems.

We observe that all methods found parameter vectors that lie in a rather small region of the search space, indicating the cost landscape near the minimum has structural stability. However, there are differences in the spread of the parameters indicating different search characteristics of the algorithms. The proportional gain $\:{K}_{p}\:$lies across the algorithms in the interval (3.18, 3.79). It is clear that TMGO obtains the maximum proportional gain, while WOA obtains the minimum. The integral gain $\:{K}_{i}$ has a more pronounced spread. In particular, eQIO gives the maximum integral gain (0.2273), suggesting emphasis on elimination of steady-state error. On the other hand, MGO and TMGO produce reasonably small $\:{K}_{i}\:$values, which means they tend to adopt more conservative integral actions. Regarding the derivative gain $\:{K}_{d}\:$, WOA and SFOA obtain the largest values, which can facilitate achieving better transient damping at the expense of being more sensitive to noise. The QIO variants prescribe less aggressive derivative gains, corresponding to a less pronounced transient response. The time-delay-associated parameter $\:\mathrm{N}$ attains the maximum value in TMGO and the minimum value in WOA, indicating distinct counteraction for the delayed dynamics. Regarding the set-point weighting factors, $\:\beta\:$and $\:\gamma\:$are similar across all algorithms. This suggests that the optimal reference-tracking structure is relatively invariant with respect to the choice of optimization method. However, small variations may influence overshoot behavior and transient smoothness.

The theoretical framework introduced in Sect.  2.3–2.5 is directly reflected in the controller tuning procedure summarized in Table 4. For each optimization algorithm, the parameter vector is encoded as a candidate solution and evaluated using the composite objective function described in Sect.  2.3. The objective function combines normalized tracking accuracy measures, transient response characteristics, steady-state error, and barrier penalties to assess the overall control performance. All algorithms employ a population size of 30 individuals and 100 iterations, resulting in a total of 3000 objective function evaluations per run. The optimized parameter sets reported in Table 4 correspond to the best solutions obtained from this procedure and are subsequently implemented without modification in the real-time heat-flow system experiments. Therefore, the experimental results presented in the following sections constitute the direct practical realization of the theoretical optimization framework developed in this study.

**Table 4:** Optimized 2DOF-PID controller parameters obtained by different algorithms.

Algorithm	$\:{\boldsymbol{K}}_{\boldsymbol{p}}$	$\:{\boldsymbol{K}}_{\boldsymbol{i}}$	$\:{\boldsymbol{K}}_{\boldsymbol{d}}$	$\:\boldsymbol{N}$	$\:\boldsymbol{\beta\:}$	$\:\boldsymbol{\alpha\:}\:$
QIO	3.4560	0.1796	0.0466	1.9463	1.2097	0.9015
MGO	3.5605	0.0947	0.0632	1.9464	1.1852	0.9195
TMGO	3.7907	0.0972	0.0515	2.0084	1.1608	0.8860
WOA	3.1823	0.1320	0.0789	1.5431	1.2288	0.9297
SFOA	3.4660	0.1089	0.0782	1.6701	1.2089	0.8950
eQIO	3.5175	0.2273	0.0491	1.7300	1.2074	0.9001

### Experimental analysis

**Fig. 8:**
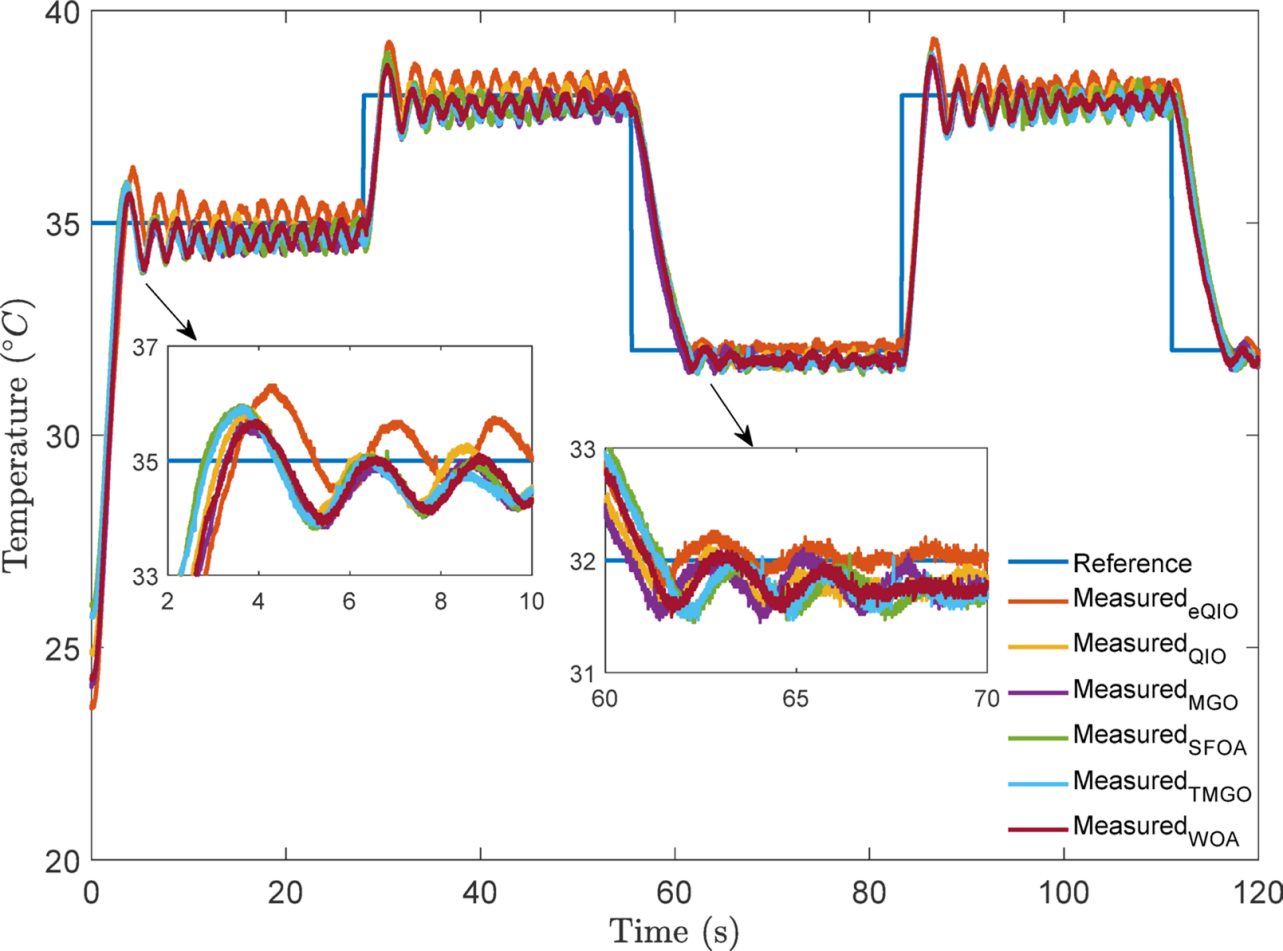
The experimental results for all optimization methods for step + square.

The Fig. 8 presents the temperature tracking performances obtained for all optimization methods for the step + square reference signal. First, in the step reference, all optimization methods quickly rose to the reference signal to capture it in the transient regime. From the figure, the fastest rise time was obtained with the TMGO and SFOA methods, approximately 2.7 s. These methods were then followed by QIO, WOA, and eQIO, respectively. On the other hand, although all optimization methods attempt to follow the reference signal in the steady-state regime, the eQIO method has followed the step reference better with the least steady-state error. However, when a square reference with sudden changes is applied, almost all methods have been found to quickly capture the reference. On the other hand, in the steady state of the square reference, the eQIO method has been found to provide better temperature tracking than the other methods, despite slight temperature fluctuations. Furthermore, in the negative alternation of the square reference, the eQIO method has almost zero steady-state error, while other methods have steady-state error. In conclusion, it was observed that the eQIO method tracked the reference temperature more successfully than other methods under step + square reference. Also, it was observed that the TMGO, WOA, MGO, and QIO methods, in particular, had steady-state error values of approximately 0.5 °C (in terms of amplitude) in the time-varying part of the reference signal.

**Fig. 9:**
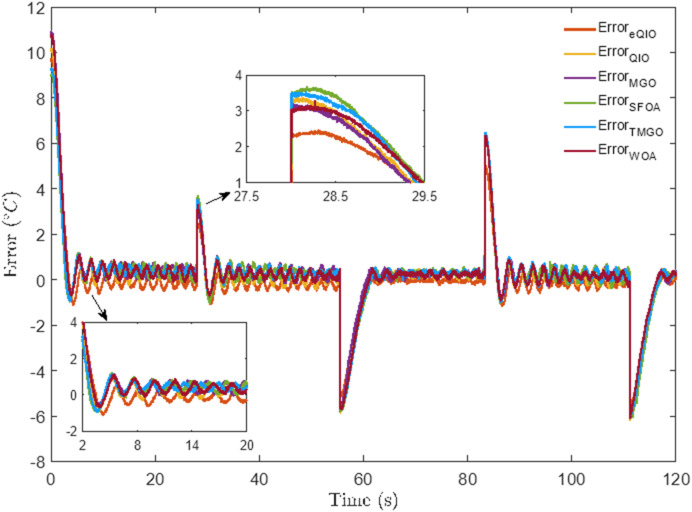
The error variations of all optimization methods under step + square.

In Fig. 9, the error suppression performance of controllers tuned using different heuristic optimization algorithms is comprehensively compared in the time domain. The results presented in Fig. 8 show that the proposed eQIO-based approach provided a significant advantage in both transient and steady-state performance compared to the QIO, MGO, SFOA, TMGO, and WOA algorithms.

**Fig. 10:**
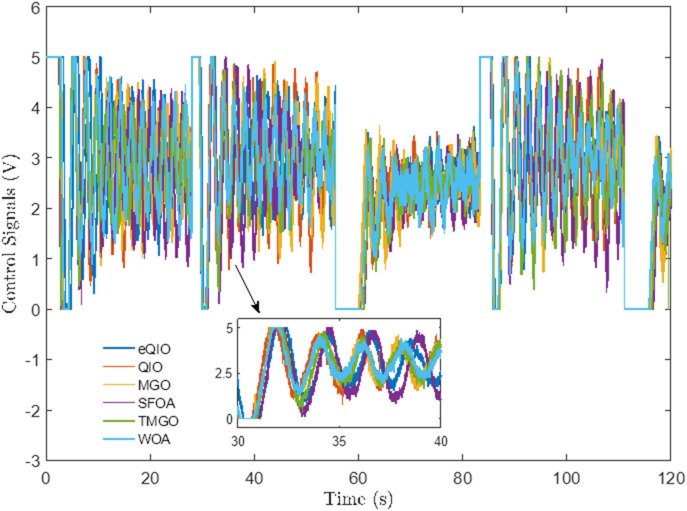
The control signal variations of all optimization methods under step + square reference signals.

Although all methods converged from high initial error to the zero-error region in a short time in the initial transient regime, the eQIO algorithm exhibited lower overshoot and better damping characteristics. While deeper negative overshoot and longer settling times were observed in the WOA and TMGO-based controllers, the eQIO approach provided a fast and smooth transition. In the steady-state region, the error amplitude of the eQIO-based controller is smaller compared to other methods, and the oscillation behaviour is significantly suppressed. It can be seen that the proposed method leads to a smaller MAE value and better steady-state performance. The QIO and WOA algorithms show moderate performance, whereas TMGO and SFOA fluctuate more and exhibit larger error variance. In addition, when the system is subject to abrupt reference changes, the eQIO-based controller achieves the lowest peak error and the fastest recovery time. This confirms that the proposed method has higher robustness and adaptability to disturbing effects. Overall, the results show that the eQIO algorithm offered more balanced performance between overshoot, settling time, and steady-state error thanks to its optimized controller parameters.

When the control signals presented in Fig. 10 are examined, it is seen that all methods produced high-amplitude control inputs in the initial transient regime due to initial error magnitude. However, the proposed eQIO-based controller exhibited a more limited control amplitude and lower variance compared to other heuristic algorithms, regulating the system dynamics in a more balanced manner. It is particularly noteworthy that the eQIO method shortened the saturation period and provided faster and smoother post-saturation recovery, especially during the time intervals when actuator saturation was observed. In contrast, longer saturation periods and significant high-frequency oscillations were observed in MGO, WOA and SFOA-based controllers. These results clearly demonstrate that the eQIO approach is superior not only in terms of error suppression performance but also in terms of control energy efficiency and providing an actuator-friendly control structure.

**Table 5:** The MAE values of all optimization methods for step + square reference signal.

Methods	eQIO	QIO	MGO	SFOA	TMGO	WOA
Step	0.8596	0.9025	1.0745	0.8966	0.9452	0.9999
Square	0.5252	0.6487	0.6865	0.7716	0.7544	0.6657
Total	0.6032	0.7079	0.7777	0.8008	0.7989	0.7437

Examining the numerical MAE results presented in Table 5, it is clearly seen that the proposed eQIO-based approach provided the lowest performance index in all test scenarios. Under the step reference signal, the eQIO method achieved the best result with a value of 0.8596, which corresponds to an improvement of approximately 4.8% compared to QIO, 9.1% compared to TMGO, and approximately 20.0% compared to MGO. In the square reference scenario, the superiority of eQIO became even more pronounced; with a value of 0.5252, it produced approximately 19.0% lower cost compared to QIO, 21.1% lower cost compared to WOA, and approximately 31.9% lower cost compared to SFOA. In the sense of the total performance index, eQIO, with a value of 0.6032, achieved an overall performance that was about 14.8%, 18.9%, and 24.7% better than its nearest rivals QIO, WOA, and SFOA, respectively. These quantitative results clearly show that the small overshoot, fast recovery, and more balanced control behaviour of the proposed controller are strongly supported not only qualitatively but also quantitatively in the error and control signal analyses.

Beyond the numerical comparison, the results reported in Table 5 provide important insights into the dynamic behaviour of the optimized controllers. The consistently lower MAE values obtained by the eQIO-based approach indicate that the proposed optimization strategy not only reduces the overall tracking error but also improves the balance between transient and steady-state performance. In the step-response scenario, the relatively small improvement over the baseline QIO suggests that both algorithms can effectively stabilize the thermal process; however, the enhanced local exploitation and stagnation recovery mechanisms of eQIO enable a more accurate adjustment of the controller parameters, resulting in lower residual steady-state error. The advantage of eQIO becomes much more evident under the square-wave reference, where the controller must repeatedly respond to abrupt set-point changes. The significant reduction in MAE under this operating condition demonstrates that the proposed algorithm provides superior adaptation to rapidly varying thermal dynamics and mitigates the accumulation of tracking errors after each reference transition. Furthermore, the lower total MAE value achieved by eQIO confirms that the controller tuning process successfully balances fast response, overshoot suppression, and steady-state accuracy without increasing control aggressiveness. This observation is also consistent with the error and control signal profiles shown in Figs. 9 and 10, where the eQIO-based controller exhibits reduced oscillatory behaviour and smoother actuator activity. In contrast, the higher MAE values obtained by MGO, TMGO, WOA, and SFOA indicate that these methods either produce larger transient deviations or require a longer time to settle around the desired temperature. Therefore, Table 5 not only demonstrates the numerical superiority of the proposed method but also provides quantitative evidence that the eQIO-tuned 2DOF-PID controller achieves a more robust and practically desirable trade-off between tracking accuracy, control smoothness, and disturbance rejection capability.

**Fig. 11:**
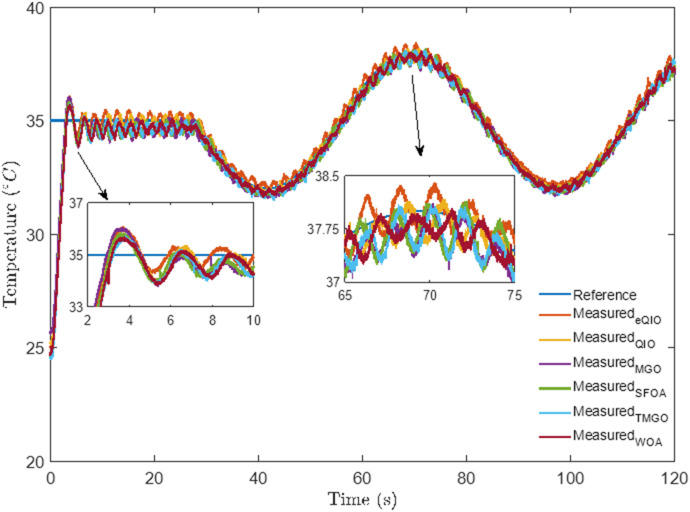
The experimental results for all optimization methods for step + sinusoidal reference signal.

By examining the temperature outputs as shown in Fig. 11, it is confirmed that the eQIO controller performs better than other controllers under all tested scenarios, including sudden changes in the reference signal, steady-state operation, and sinusoidal tracking conditions. Transiently, eQIO attains the reference rather fast with an overshoot of about 36 °C; on the contrary, the overshoot of WOA- and MGO-based approaches is above 36 °C, with oscillations persisting further in time. In the steady-reference domain, the temperature oscillations of eQIO are around ± 0.2 °C, while those of QIO, TMGO, and WOA go beyond ± 0.3–0.85 °C. The eQIO solution maintains the error between the reference and the peak, and between the reference and the bottom, within ± 0.1–0.3 °C during sinusoidal reference tracking, and it has the least amplitude error and phase delay, whereas the TMGO- and MGO-based solutions have errors larger than 0.4–0.5 °C. These results confirm that eQIO leads to reduced overshoot, smaller steady-state ripple, and better dynamic tracking in different operating modes, and demonstrate that the designed controller represents a more balanced and more robust control scheme consistent with the previous error, control signal, and numerical performance results.

**Fig. 12:**
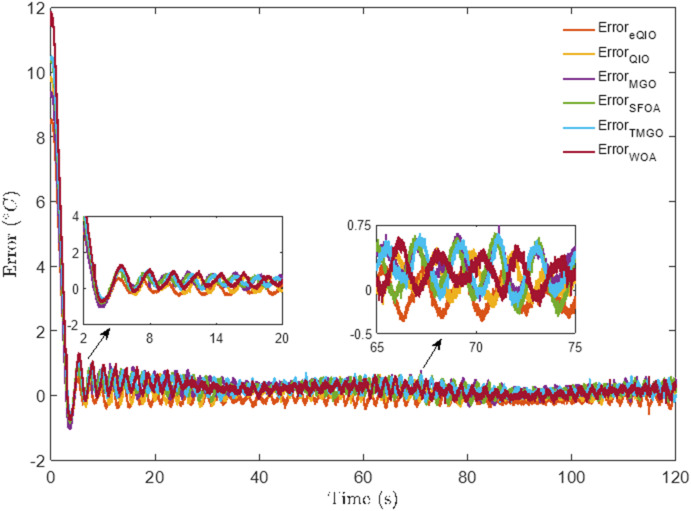
The error signal variations of all optimization methods under step + sinusoidal reference signals.

When comparing the error dynamics shown in Fig. 12, it can be seen that all approaches eliminate the large initial error of around 10–12 °C in a short time, but noticeable differences arise in terms of convergence speed and damping behavior. In the early transient mode, the eQIO-based controller provides a well-damped response with a small negative undershoot of about − 1.5 °C, quickly moving the error around zero.

Conversely, in the WOA- and MGO-based schemes, deeper overshoots and longer oscillations arise. After the preliminary settling (≈ 5–20 s), the eQIO error magnitude is constrained to about ± 0.4 °C, whereas this value reaches ± 0.45–0.75 °C in QIO and TMGO, and ± 0.9 °C in WOA. In the long-term steady-state range (20–120 s), although small periodic fluctuations are observed in all methods, the eQIO method exhibits the minimum error variance and the smoothest error profile. Particularly in the zoomed-in region of 65–75 s, the error magnitude of eQIO remains around ± 0.35 °C, while it increases from − 0.2 to 0.7 °C in the WOA and MGO methods. These results further indicate that the eQIO-based controller achieves faster and more damped error reduction in the transient region and lower steady-state error together with better disturbance rejection in steady state. Overall, the error graph, consistent with the control signal and performance index results, confirms that the eQIO approach provided more balanced, stable, and reliable control performance compared to other heuristic optimization-based methods.

**Fig. 13:**
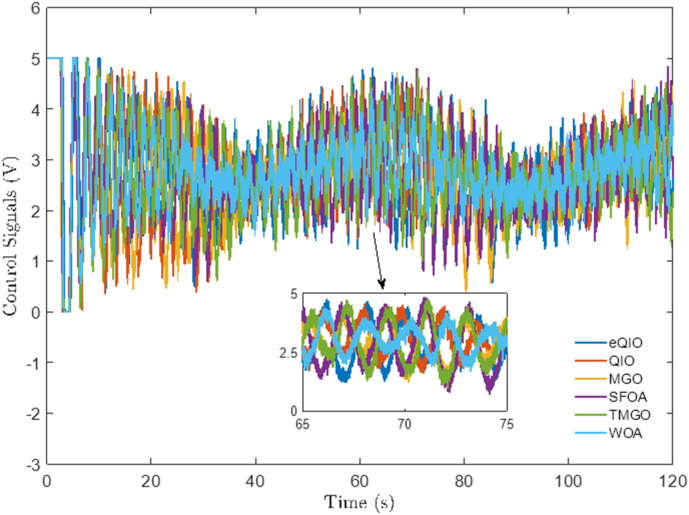
The control signal variations of all optimization methods under step + sinusoidal reference signals.

When the control signal behaviours presented in Fig. 13 are examined, it is seen that all methods have produced high-amplitude and rapidly changing control signals at the initial stage. This is due to the attempt to suppress the large initial error in the system in a short time. However, significant differences were observed in terms of how the controllers generate the control signal and their aggressiveness levels. The eQIO-based controller, while largely keeping the control signals in the 0–5 V operating range, exhibited a more stable control profile compared to other methods, especially after the transient regime, showing more limited amplitude and lower variance. In contrast, in the WOA and MGO-based methods, the control signals were more irregular, with sudden peaks and high-frequency oscillations occurring more frequently. In the medium and long-term steady-state region (approximately 40–120 s), the control signal amplitude of the eQIO approach followed a smoother control signal magnitude in the range of approximately 2–4 V, while in the QIO, SFOA, and TMGO methods, this range was observed to be wider and the chattering level increased. Overall, the control signal analysis, consistent with the error and tracking performance, confirms that the eQIO approach provided a more balanced, stable control structure that is more suitable for real-world system applications in which the optimization-based control is applied. The numerical results presented in the Table 6 clearly demonstrate a comparative performance of different methods under Step + Sinusoidal input. Under Step input, the lowest MAE value was obtained with the eQIO method (0.6883), which exhibited the fastest and most stable response to sudden reference changes. On the other hand, eQIO was followed by QIO (0.9024) and MGO (0.9437), while SFOA (0.9999), WOA (0.9896), and especially TMGO (1.0202) showed relatively weaker performance with higher MAE values. In the case of sinusoidal input, the error values decreased significantly in all methods, with the best result obtained by eQIO (0.1322), followed by WOA (0.1521) and QIO (0.1612). This shows that the eQIO has a very strong ability to track periodically and continuously changing references.

**Table 6:** The MAE values of all optimization methods for step + sinusoidal reference signal.

Methods	Step	Sinusoidal	Step + Sinusoidal
eQIO	0.6883	0.1322	0.1692
QIO	0.9024	0.1612	0.3119
MGO	0.9437	0.2491	0.5364
SFOA	0.9999	0.1978	0.3850
TMGO	1.0202	0.2067	0.5172
WOA	0.9896	0.1521	0.4533

Finally, when the MAE values obtained throughout the entire reference signal were examined, eQIO (0.1692) maintained its superiority by offering the lowest error value by far, while QIO (0.3119) and SFOA (0.3850) showed moderate performance, and MGO (0.5364), TMGO (0.5172), and WOA (0.4533) struggled to adapt to complex dynamics with higher error values. Overall, in all test scenarios, the eQIO method demonstrated a more balanced, stable, and superior control performance compared to other methods, thanks to its lower error output against both sudden and periodic reference changes.

Furthermore, the obtained results also confirm the practical impact of the theoretical improvements introduced in the proposed eQIO algorithm. The periodic local refinement mechanism contributes to a more accurate adjustment of the controller gains, while the stagnation-aware diversity recovery strategy helps avoid premature convergence during optimization. As a consequence, the eQIO-tuned controller exhibits a balanced trade-off between fast transient response, low overshoot, reduced steady-state error, and moderate control effort. The consistency of these observations across both the step + square and step + sinusoidal reference scenarios further demonstrates the robustness and reliability of the proposed optimization framework.

Overall, the proposed eQIO-based 2DOF-PID strategy offers several advantages, including improved convergence reliability, enhanced tracking accuracy, reduced steady-state error, and smoother control action. However, these benefits are achieved at the expense of a moderate increase in computational effort during the offline optimization stage due to the additional local search and stagnation-handling mechanisms. Moreover, the controller performance depends on the selected objective function and may require re-optimization if the operating conditions or system dynamics change significantly. Therefore, the proposed method represents a trade-off between optimization robustness and computational complexity, making it particularly suitable for applications where offline controller tuning is acceptable.


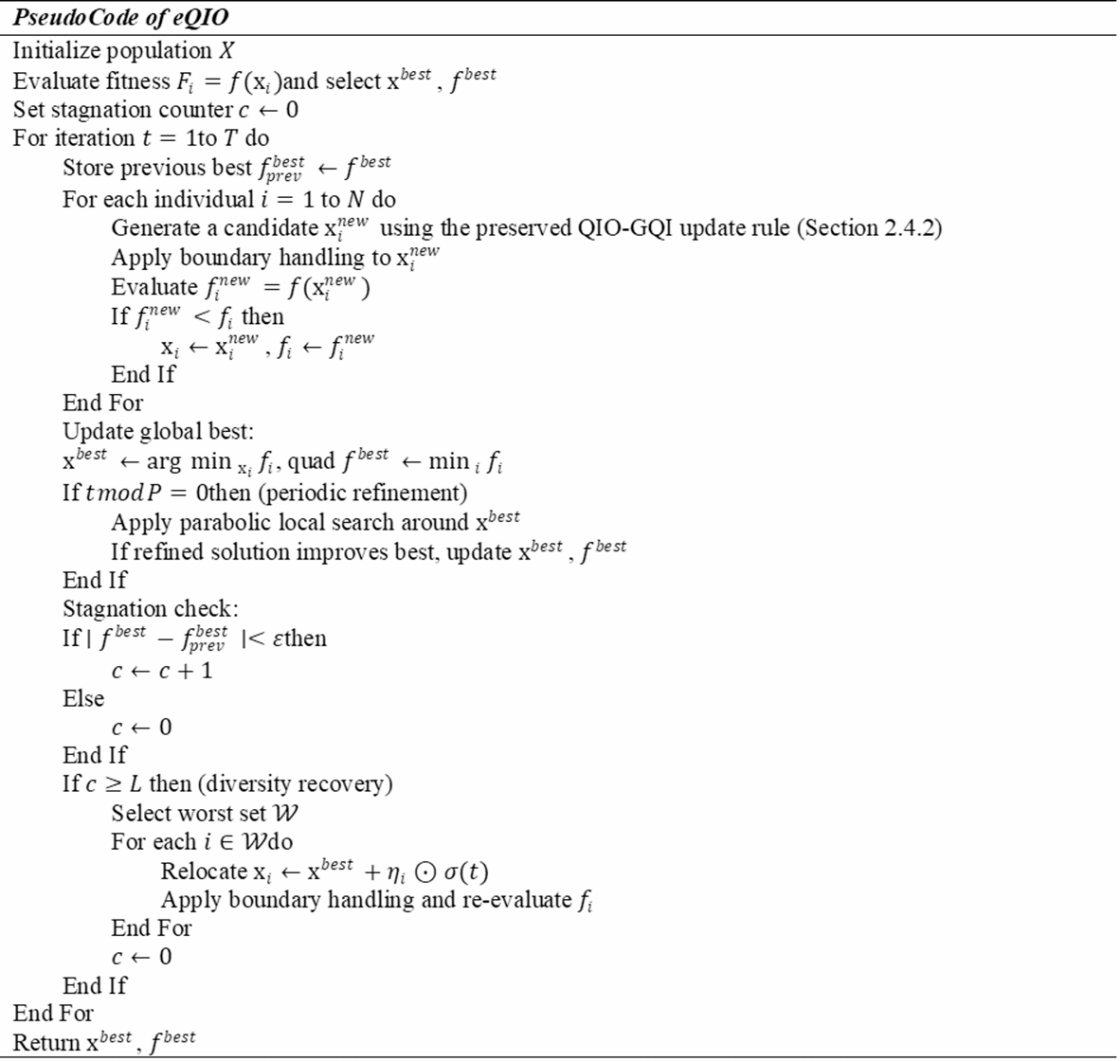


### Limitations and practical considerations

The proposed study focuses on the optimal control of a heat flow system through an eQIO-tuned 2DOF-PID controller. While the results demonstrate improved control performance and enhanced optimization robustness, several limitations and practical considerations should be addressed.

First, the optimization process is carried out offline. As in the case of the baseline QIO, the eQIO algorithm also includes additional mechanisms, namely periodic local search and stagnation handling, which increase the number of objective function evaluations. While this has no impact on real-time controller operation after the parameters have been assigned, it does introduce additional computational cost during the tuning process. This computational cost should be further investigated, especially for systems with a high number of states and/or for systems that require frequent retuning or adaptive online optimization. Second, the performance of the optimized controller is sensitive to the selected performance index and the imposed parameter constraints. Alternative cost function formulations may result in different parameter values and distinct transient behaviour. In other words, extrapolating the obtained parameter set to other thermal systems or operating scenarios should be done with care. In practical applications, when system dynamics or operating conditions change, re-optimization may be required. Finally, the heat flow apparatus is studied with real-time experiments rather than only with simulation in this work. Although this indicates that the presented methodology may be applicable, long-term testing under multiple operating conditions would validate its robustness. Since the 2DOF-PID structure allows independent tuning of reference tracking and disturbance rejection via the weighting factors $\:\beta\:$ and $\:\alpha\:,$ it provides more degrees of freedom for this particular application.

To conclude, a single-objective optimization problem is solved in this paper. In most applications of thermal control, several competitive goals should be fulfilled at the same time. Hence, it would be interesting to consider the improved QIO-based methodology for multi-objective formulations, which is left as future work. Although these disadvantages, the eQIO-based 2DOF-PID methodology is still a feasible method to improve the performance of heat flow regulation. The structured control system design in combination with a robust optimization approach is particularly well suited for thermal systems.

## Conclusıon

This paper deals with a 2DOF-PID controller for a heat flow process, and the proposed controller is optimally tuned based on the improved eQIO algorithm. The proposed modification of the standard quadratic interpolation scheme consists in introducing periodic local refinement and stagnation-aware diversity recovery to increase overall convergence robustness. During the real-time heat flow control experiments, two reference scenarios are considered: step + square and step + sinusoidal. For the step + square reference, the eQIO-based controller achieves approximately 4.8% lower MAE in the step component, 19.0% lower MAE in the square-wave component, and 14.8% lower total MAE compared with the baseline QIO-based controller. For the step + sinusoidal reference, the eQIO-based controller provides approximately 23.7% lower MAE in the step component, 18.0% lower MAE in the sinusoidal component, and 45.8% lower total MAE relative to QIO. These quantitative results indicate that the proposed eQIO-based tuning strategy yields substantially improved tracking accuracy and overall error suppression across both abrupt and continuously varying reference signals. The numerical findings are consistent with the time-domain response plots, where the eQIO-based controller exhibits reduced oscillatory behavior, faster recovery after reference changes, and smoother control action compared to the competing methods. Overall, the results confirm that the proposed eQIO-based 2DOF-PID scheme delivers improved optimization reliability and enhanced thermal control performance without increasing algorithmic complexity.

## Data Availability

All data generated or analysed during this study are included in this published article.

## Code availability

The reference Python implementation of the proposed Enhanced Quadratic Interpolation Optimization (eQIO) algorithm is publicly available at Zenodo: 10.5281/zenodo.21256121.
